# Impact of microRNAs on regulatory networks and pathways in human colorectal carcinogenesis and development of metastasis

**DOI:** 10.1186/1471-2164-14-589

**Published:** 2013-08-29

**Authors:** Silvia Pizzini, Andrea Bisognin, Susanna Mandruzzato, Marta Biasiolo, Arianna Facciolli, Lisa Perilli, Elisabetta Rossi, Giovanni Esposito, Massimo Rugge, Pierluigi Pilati, Simone Mocellin, Donato Nitti, Stefania Bortoluzzi, Paola Zanovello

**Affiliations:** 1 Oncology and Immunology Section, Department of Surgery, Oncology and Gastroenterology, University of Padova, Padova, Italy; 2 Department of Biology, University of Padova, Padova, Italy; 3 Istituto Oncologico Veneto (IOV), IRCCS, Padova, Italy; 4 Surgical Pathology and Cytopathology Unit, Department of Medicine, University of Padova, Padova, Italy; 5 Surgery Section, Department of Surgery, Oncology and Gastroenterology, University of Padova, Padova, Italy

**Keywords:** microRNA, Gene expression, Regulatory networks, Colorectal cancer, Metastasis

## Abstract

**Background:**

Qualitative alterations or abnormal expression of microRNAs (miRNAs) in colon cancer have mainly been demonstrated in primary tumors. Poorly overlapping sets of oncomiRs, tumor suppressor miRNAs and metastamiRs have been linked with distinct stages in the progression of colorectal cancer. To identify changes in both miRNA and gene expression levels among normal colon mucosa, primary tumor and liver metastasis samples, and to classify miRNAs into functional networks, in this work miRNA and gene expression profiles in 158 samples from 46 patients were analysed.

**Results:**

Most changes in miRNA and gene expression levels had already manifested in the primary tumors while these levels were almost stably maintained in the subsequent primary tumor-to-metastasis transition. In addition, comparing normal tissue, tumor and metastasis, we did not observe general impairment or any rise in miRNA biogenesis. While only few mRNAs were found to be differentially expressed between primary colorectal carcinoma and liver metastases, miRNA expression profiles can classify primary tumors and metastases well, including differential expression of miR-10b, miR-210 and miR-708. Of 82 miRNAs that were modulated during tumor progression, 22 were involved in EMT. qRT-PCR confirmed the down-regulation of miR-150 and miR-10b in both primary tumor and metastasis compared to normal mucosa and of miR-146a in metastases compared to primary tumor. The upregulation of miR-201 in metastasis compared both with normal and primary tumour was also confirmed. A preliminary survival analysis considering differentially expressed miRNAs suggested a possible link between miR-10b expression in metastasis and patient survival. By integrating miRNA and target gene expression data, we identified a combination of interconnected miRNAs, which are organized into sub-networks, including several regulatory relationships with differentially expressed genes. Key regulatory interactions were validated experimentally. Specific mixed circuits involving miRNAs and transcription factors were identified and deserve further investigation. The suppressor activity of miR-182 on ENTPD5 gene was identified for the first time and confirmed in an independent set of samples.

**Conclusions:**

Using a large dataset of CRC miRNA and gene expression profiles, we describe the interplay of miRNA groups in regulating gene expression, which in turn affects modulated pathways that are important for tumor development.

## Background

The dysregulation of microRNA (miRNA) expression in tumors compared with normal counterparts has been observed in many hematologic and solid tumors. Several miRNAs have been proposed to act as tumor-suppressor or tumor-promoting genes [1], and specific miRNA expression signatures with potential prognostic significance have been observed in various primary tumors analysed so far, including colon, lung, pancreatic cancer and neuroblastoma. Interestingly, pre-malignant lesions (such as adenomas) share identical alterations in miRNA expression with carcinoma, suggesting that the acquisition of cancer-specific profiles represents an early event in the malignant process [2].

The role of miRNAs in metastasis development is less clearly defined, and contradictory results in this field have been reported, depending on the experimental system studied, the cellular and tissue context, and the step of the metastatic process analysed. A number of so-called oncomiRs have been identified for their ability to influence key steps in the metastatic process directly [3-5]. In some cases, sets of metastasis-associated miRNAs different from those involved in tumorigenesis have been reported [6]. For instance, specific miRNAs are involved in circuits regulating the epithelial to mesenchymal transition (EMT), a critical step which drives tumor metastasis [7]. The major limitation of these studies is that functional data have mainly been collected by means of *in vitro* assays with tumor cell lines, and only a limited number of studies have been carried out *in vivo*.

Colorectal cancer (CRC) is a leading cause of cancer-related mortality in the USA and Europe [8,9]. Improved treatment strategies involving surgery, chemo- and radiotherapy have increased the overall survival rates in early stages, but tumor recurrence (particularly in lymph-node-positive cancers) is frequent. About one-third of CRC patients develop synchronous or metachronous metastases in the liver. The 5-year overall survival rate of patients with CRC decreases from 80-90% in the case of locally confined tumors, to 40-60% in locally advanced non-metastatic tumors, and to only 5-10% in metastatic tumors [10]. Over the last few years, various miRNA expression patterns observed in primary colorectal tumors have been associated with tumor stage and patient survival [11-13], while only few miRNAs are common to all reported expression profiles. An interesting example is miR-21, high levels of which in CRC compared with normal colon tissue have been associated with poor prognosis and unfavorable therapeutic response, independently of well-established clinical predictors [14,15]. In the same studies, other differentially expressed miRNAs, i.e., miR-20a, miR-106a, miR-181b, miR-203 and miR-143, were analysed for their prognostic value, but the strength of their association with poor survival was less robust. Although clinical and pathological parameters are available for the prognostic stratification of CRC patients, more comprehensive knowledge of the basic features of colorectal tumorigenesis and metastatic process may have important implications for both scientific and clinical research and could help in answering a variety of long- standing questions. For instance, the frequency of epigenetic and transcriptional changes occurring in primary versus metastatic lesions is still an open issue.

In this study, we performed a genome-wide expression analysis of both miRNA and genes in primary tumors, liver metastases and normal colon mucosa from CRC patients, with the aim of discovering modulated miRNAs and genes in tumor and metastasis development and to identify the regulatory networks involved in tumor progression.

## Results

### Matched miRNA and gene expression in normal colon mucosa, primary colon carcinoma and liver metastasis

We determined the miRNA expression profiles of 78 samples (23 normal colon mucosa, N; 31 primary colon carcinoma, T; and 24 liver metastases, M), obtained from 46 patients (Table 1). This dataset included 24 samples belonging to 8 patients with matched samples (N, T and M) from the same patient. To understand how changes in miRNA expression can influence gene expression, we also examined the expression profiles of 22,517 genes in 80 samples, including 23 N, 30 T and 27 M, and comprising 27 matched samples from 9 patients (Table 2). Data are available at the GEO database (GSE35834).

**Table 1:** Patient data

**Characteristics**	
**No of patients (n)**	46
**Age (years, mean ± s.d.)**	60.7 ± 10.2
**Gender**	
Female	17
Male	29
**Tumor site**	
Cecum, colon ascending, transverse colon	13
Splenic [left] flexure, colon descending, sigmoid colon	20
Rectum	13
**TNM stage**	IV
**Liver metastasis**	
Synchronous	39
Metachronous	7

**Table 2:** Sample set description for miRNA and gene array datasets

**Array**	**Match type**	**Number of patients**	**Tissue type**	**Number of samples**
**miRNA**	N-T-M	8	N	23
	N-T	7		
	T-M	8	T	31
	M-N	2		
	N	6	M	24
	T	8		
	M	6	Total	78
	Total	45		
**Array**	**Match type**	**Number of patients**	**Tissue type**	**Number of samples**
**Genes**	N-T-M	9	N	23
	N-T	5		
	T-M	8	T	30
	M-N	3		
	N	6	M	27
	T	8		
	M	7	Total	80
	Total	46		

For both miRNA and gene array samples, column 3 shows number of patients for whom paired data for various tissue type combinations were available; last column reports total number of samples for each tissue type.

Of 847 miRNAs represented in the array, 309 were considered for later analyses after filtering (see Methods). Similarly, 30% of genes with low expression profile variability were filtered out, and 15,761 genes with moderately to highly variable expression profiles were considered further.

**Figure 1:**
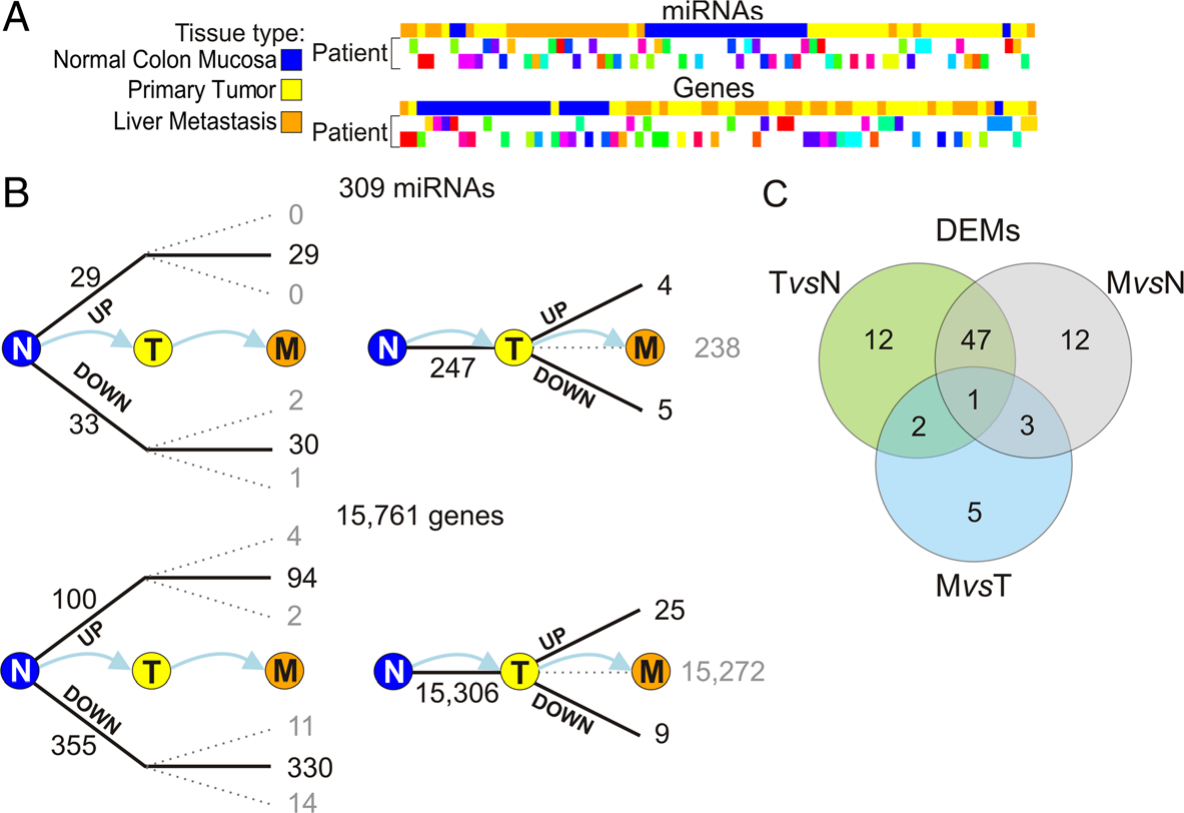
**miRNAs and gene expression in normal colon mucosa, primary tumor and liver metastases. (A)** Sample classification based on 309 miRNAs and 15,761 gene expression profiles. Color-coding of samples reported in different lines refers to tissue type (Normal colon mucosa, N; primary colorectal cancer, T and liver metastasis, M) and per-patient matching of samples. **(B)** miRNA and gene expression variability in two main tumor progression transitions. **(C)** Venn diagram of intersections among DEMs identified by unpaired test applied to different comparisons.

Unsupervised hierarchical cluster analysis was performed on selected miRNAs and genes. Normal samples clustered together and were relatively well separated from T and M samples, both according to unsupervised hierarchical cluster analysis based on miRNA and on gene expression data (Figure 1A). In fact, only miRNA expression profiles separated T from M quite well. Considerable per-patient pairing of T and M samples was observed in both dendrograms, in which triplets and pairs of samples from the same patient are shown in the same color (see also Additional file 1: Figure S1). In roughly 25% of patients, the M samples were more similar to the T from which they had derived, rather than to the M samples of other patients. The similarity between T and M samples of the same patient was more evident when samples were classified according to gene expression (20% and 28% of per-patient sample pairing, in miRNA- and gene-based heatmaps, respectively).

miRNA expression variability in the two main transitions in the N-T-M progression was evaluated, by considering the number of miRNAs and genes resulting up- or down modulated (absolute fold-change > 1) or unchanged in the T *vs* N and in the M *vs* T comparisons. Figure 1B shows that most expression variation took place in the T *vs* N comparison, revealing two alternative patterns of miRNA (and gene) expression variation in the progression: i) miRNAs (genes) up- or down-modulated in the comparison T *vs* N and basically stable in M *vs* T (Figure 1B, left), and ii) miRNAs (genes) unchanged in T *vs* N and modulated in M *vs* T (Figure 1B, right).

### Differentially expressed miRNAs

Many significantly differentially expressed miRNAs (DEMs) can be found during tumor progression. The whole set of samples with miRNA expression data was considered in unpaired tests (e.g., all T *vs* all N samples). Other sample subsets matched by patient were considered for paired comparisons (Additional file 1: Methods and Results and Additional file 1: Figure S2).

By considering the larger unpaired dataset, we identified 62, 63 and 11 DEMs in T *vs* N, M *vs* N and M *vs* T comparisons, respectively (Additional file 1: Table S1). Several miRNAs were significantly modulated in more than one contrast. Only 5 miRNAs varied merely when M and T samples were compared (miR-146a, miR-15a, miR-15b, miR-196a, miR-708). Of 53 DEMs shared by at least two comparisons, 25 were always under- and 26 over-expressed, whereas two miRNAs did not follow the same trend in the various comparisons. miR-100 and mir-99a, both putative tumor suppressors, were under- expressed in T *vs* N and over-expressed in M *vs* T.

Identification of DEM confirmed that more miRNAs are modulated in T *vs* N than in M *vs* T comparison; however, DEMs in metastasis compared with primary tumors may be of great importance, since they include miR-10b, miR-210 and miR-708, which are key regulators of several processes related to disease progression, such as DNA repair, angiogenesis, hypoxia, EMT induction, and cancer recognition by the immune system [16-18]. Additional file 1: Figure S3 shows the expression profiles of 22 miRNAs involved in EMT which were differentially expressed in T *vs* N and/or M *vs* T comparisons.

### miRNA biogenesis is not impaired during cancer progression

According to our results, during tumor progression we could not detect either general impairment of miRNA biogenesis or an overall increase in miRNA expression. In fact, in both T *vs* N and/or M *vs* T contrasts, we observed a comparable number of up- and down-modulated DEMs: 29 and 33 were respectively up- and down-modulated in T *vs* N; as against 5 and 6 in M *vs* T. Regarding global miRNA expression, when we considered the distribution of all DEM expression levels measured in N, T and M, again we could not find any significant differences between the groups (mean values 6.10, 5.95 and 6.01, respectively; p-value of pairwise mean equality t-test >0.7).

### qRT-PCR validation of miRNA expression

To assess the reproducibility of the identified miRNAs, we used qRT-PCR to measure the expression of 5 miRNAs, both in the same 78 samples used for miRNA expression profiling and in an independent set of 21 samples obtained from matched samples of 7 patients. We quantified three down-regulated (miR-150, miR-10b, miR-146a) and two up-regulated miRNA (miR-210 and miR-122). miR-150 was the most down-regulated miRNA in the T *vs* N comparison; miR-10b was significantly down-regulated in all three contrasts; miR-122 and miR-146a were differentially expressed in the M *vs* T comparison; and miR-210 was up-regulated in the M *vs* T and M *vs* N comparisons. For each of the above miRNAs, the Spearman rank correlation test between qRT-PCR expression estimation (${2{-}^{∆C}}_{t}$) and array-based expression level, in the same 78 samples, was significant (p < 0.01) (Additional file 1: Figure S4A). miR-150 and miR-146a were also tested on an independent set of 21 samples (N, T and M samples from 7 new patients) and the results were fully consistent with microarray-based observations, confirming miR-150 down-regulation in T *vs* N and miR-146a down-regulation in M *vs* T (Additional file 1: Figure S4B).

The expression level of miR-122, a miRNA which is highly expressed in the liver, was significantly higher in M than in T, suggesting the residual presence of normal liver tissue in metastasis samples. miR-122 expression was evaluated in samples in which normal colon mucosa and liver tissue had been mixed in different proportions. qRT- PCR analysis showed that mixing up to 95% of N with 5% of liver tissue caused a significant increase in miR-122 expression, compared with samples with 100% of N tissue. This result indicates that the presence of only 5% of liver cells in a sample significantly increases miR-122 expression.

### Identification of most probable miRNA targets and definition of regulatory networks modulated in development of tumor and metastasis

We obtained a set of 77 samples (23 N, 29 T, 25 M) with matched miRNAs and gene expression data from the same biological sample. The combined analysis of target prediction and expression of 305 miRNAs with predicted targets and 12,748 target genes allowed us to reconstruct post-transcriptional regulatory networks describing the most probable regulatory interactions and circuits active in transitions characterizing tumor development and progression.

miRNAs interact in several ways with target mRNAs, and may exert non-canonical regulatory actions, but they commonly act post-transcriptionally by altering the stability of target mRNAs. The expression profile of a given miRNA was therefore expected to be inversely correlated with that of its target genes. To identify the most probable miRNA targets, we first adopted a classical approach, to enrich a large set of predicted miRNA targets in truly regulated genes, by identifying significant negative correlations between miRNAs and predicted target expression profiles [19,20]. With the selected threshold on correlation significance (see Methods), we identified 3,078 relations, involving 117 miRNAs and 1,423 target genes (correlation value < −0.412). It should be noted that less than 1 out of 1,500 miRNA/target relations predicted by miRSVR turned out to be supported by expression data. Indeed, among supported relationships, 2,690 (87%) were based on predicted target sites, which are conserved across species, whereas only one-quarter of the original predictions involved conserved sites (see Methods).

The number of supported target genes per miRNA ranged from 1 to 216, with an average value of 26.3. About half the genes were supported targets of only one specific miRNA, whereas other genes were putatively regulated by up to 10 different miRNAs.

Within the whole group of supported target genes, of particular importance are the various subsets of genes significantly differentially expressed (DEGs), which may be viewed as the prominent effects of post-transcriptional regulatory action exerted by miRNAs.

**Figure 2:**
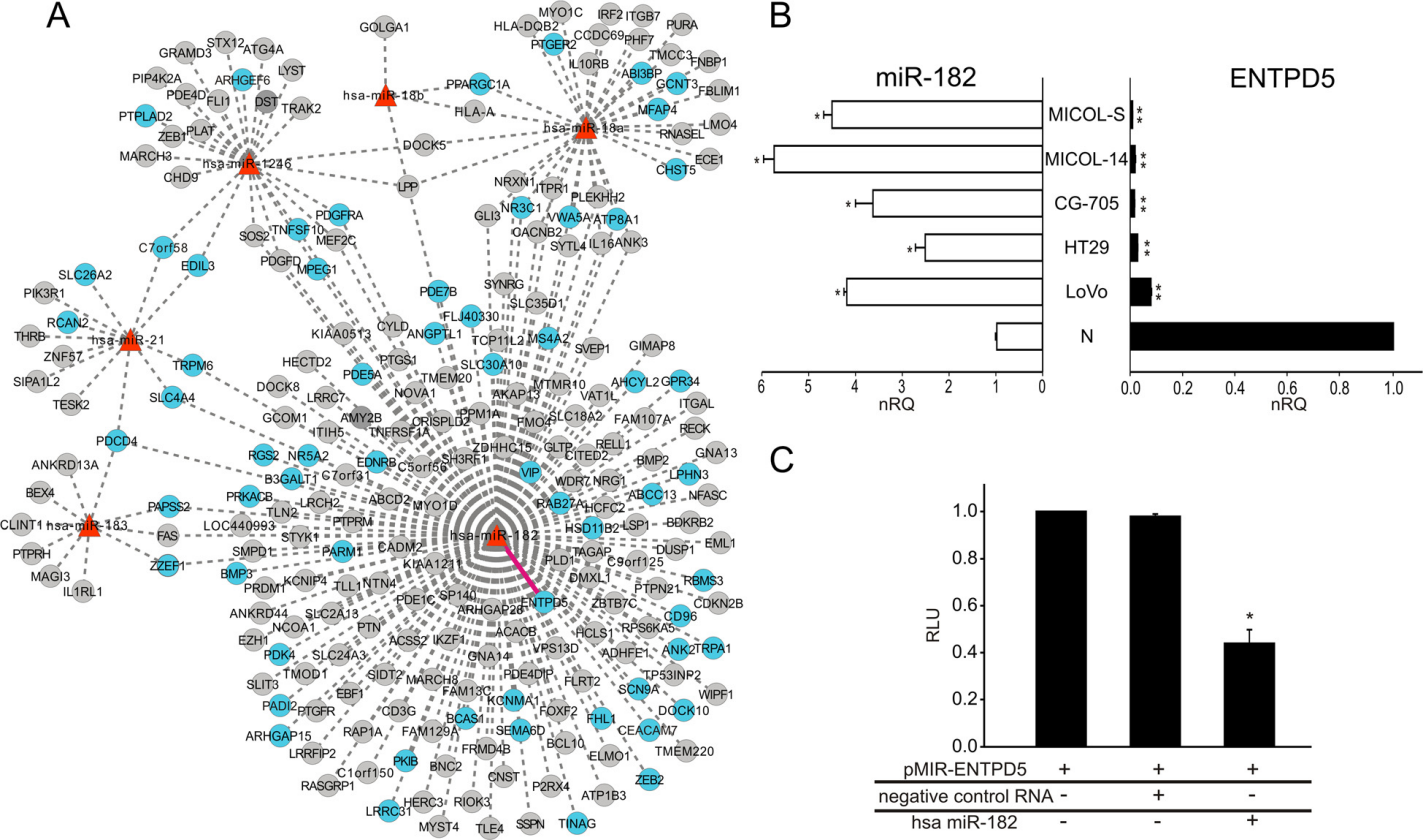
**Post-transcriptional regulatory network of miRNAs up-modulated in T (primary tumor) *vs *N (normal colon mucosa) contrast. (A)** The bipartite network represents DEMs up-modulated (FC > 3) in T *vs* N comparison (red triangles), supported target genes (circles) and their relations (gray dotted lines). Target DEGs in T *vs* N contrast are shown in blue, other genes in grey. The pink solid line outlines the experimentally validated miR-182/ENTPD5 relation. **(B)** Inverse correlation between miR-182 and ENTPD5 expression, according to qRT-PCR in 5 colon cancer cell lines and a pool of normal tissue. **(C)** Luciferase reporter assay of 3′UTR region of ENTPD5 and miR-182. Average relative light units (RLU) of biological replicates compared with control (HEK293T pMIR-ENTPD5), non-target RNA (HEK293T pMIR-ENTPD5 non-target RNA) and miR-182 over-expression (HEK293T pMIR-ENTPD5 miR-182). Data shown as means ± standard deviation (SD) of mean of three experiments performed in triplicate. * *P* < 0.05 *vs* N or control. **P < 0.01 *vs* N or control. nRQ: normalized Relative Quantity.

**Figure 3:**
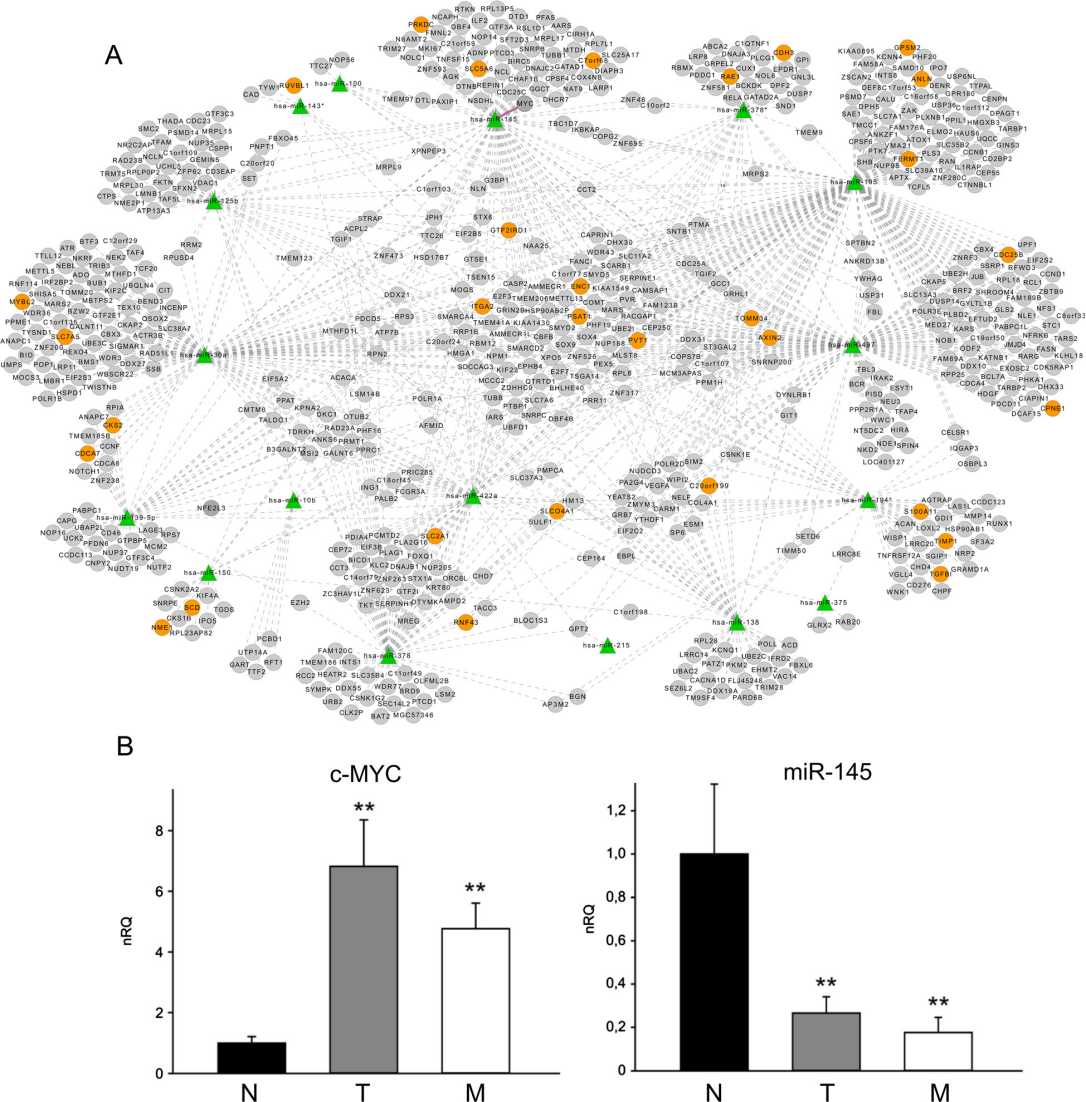
**Post-transcriptional regulatory network of miRNAs down-modulated in T *vs *N contrast. (A)** The bipartite network represents DEMs down-modulated (FC > 3) in T *vs* N comparison (green triangles), supported target genes (circles) and their relations (gray dotted lines). Target DEGs in T *vs* N contrast are shown in orange, other genes in grey. The pink solid line outlines an experimentally validated relation. **(B)** Inverse correlation between c-Myc and miR-145 expression according to quantitative qRT-PCR in 78 samples of N, T and M samples used for gene profiling. Quantification normalized to expression of DACT1 and miR-200c, respectively. Data shown as means ± standard deviation (SD) of mean of three experiments performed in triplicate. **P < 0.01 *vs* N. nRQ: normalized Relative Quantity.

As previously stated, in the unpaired analysis only 5 miRNAs appeared to be significantly modulated in the transition from primary tumor to metastasis, whereas the number of DEMs observed in the comparison between normal tissue and primary tumor and metastasis was higher (Figure 1C). It is reasonable to assume that DEMs, being moderately to highly expressed in all the considered samples and those most variable in each contrast, are responsible for most target repression. Thus, for the T *vs* N and M *vs* N comparisons, we focused only on DEMs up- and down-regulated in each contrast with a fold change (FC) > 3 and with an average expression over background in at least one of the two contrasted groups. The intersection between the post- transcriptional network and the results of miRNA differential expression analysis induced various sub-networks, describing post-transcriptional regulatory circuits involving those miRNAs whose expression variation may be important for tumor development and progression. Figures 2A and 3A show post-transcriptional regulatory networks with miRNAs differentially expressed in the T *vs* N comparison and their supported relations with target genes. Two network components are observed, involving respectively 6 up-regulated (Figure 2A) and 17 down-regulated (Figure 3A) DEMs. The component regarding the 6 up-regulated miRNAs was smaller, and a large fraction of genes appeared to be regulated by miR-182. The largest component involved 17 down-regulated DEMs, which putatively regulated a number of targets about twice as high as that observed for the whole set of miRNAs. A large proportion of the predicted target genes were indeed significantly differentially expressed. This was more evident for the up-regulated miRNA component, in which 62 down- regulated DEGs accounted for 27% of the supported targets of up-regulated DEMs. Conversely, our analysis predicted that only a few genes would be targets of more than one up-regulated miRNA. Among the interactions predicted between miRNA and target genes identified in the T *vs* N regulatory network we considered the relationship between miR-145 and c-Myc. qRT-PCR analysis of the entire set of samples confirmed the opposite behavior of miR-145 and c-Myc in tumour progression (Figure 3B).

The network involving five DEMs observed in the M *vs* T comparison and their supported target genes was small and consisted of five unconnected components (Additional file 1: Figure S5). No DEGs were observed among the supported targets of DEMs in the T to M transition. These results further support the concept that the main change in the transcriptome occurs early in the CRC progression.

### miR-10b expression in metastases: potential association with survival

The 26 miRNAs included in the TN and MT networks (Additional file 1: Table S3) were considered for survival analysis. The expression of miR-10b measured in liver metastasis showed a statistically significant association with the survival of patients affected with stage IV CRC (hazard ratio = 1.47, 95% confidence interval = 1.23-1.75; adjusted p-value: 0.00052). The effect of miR-10b on prognosis is given in Additional file 1: Figure S6, which shows that patients with high levels of miR-10b expression in their metastatic disease have a shorter time to event (median survival: 8 months) compared with those with low levels (51 months). In our study, the expression levels of miR-10b measured in primary tumors had no significant impact on prognosis.

### miR-182 controls ENTPD5

miR-182 was one of the most upregulated DEMs in the T *vs* N contrast. Among its predicted target genes we focused on ENTPD5, due to the involvement of the gene product in energy metabolism. Since ENTPD5 resulted significantly down-regulated in our analysis, and another study provided support for downregulation during cancer progression [21], we decided to study the relationship between miR-182 and its predicted target gene ENTPD5. To investigate the opposite behavior of the miRNA and its target gene, we performed qRT-PCR to measure miR-182 and ENTPD5 expression in a panel of five cell lines (CG-705, HT29, from a primary colorectal tumor, and MICOL-S, MICOL-14, and LoVo, from colorectal carcinoma metastases [22]). As shown in Figure 2B, all tumor cell lines showed an inverse correlation (−0.85, p-value < 0.05) between high expression of miR-182 and low expression of ENTPD5. This result not only confirms the microarray profiling data but also suggests a role of the anti-correlated relationship in conferring some advantageous properties to the tumor cells. We next wanted to provide support to the direct targeting of ENTPD5 by miR-182. To this end, we performed a Luciferase reporter assay in HEK293T cells transfected with a construct containing the firefly luciferase gene fused to the 3′-UTR of ENTPD5 (pMIR-ENTPD5). When cells were co-transfected with miR-182, a 50% reduction in luciferase expression was observed, compared with cells transfected with negative control RNA or pMIR-ENTPD5 only (Figure 2C).

### miRNA modulated KEGG pathways

For the T *vs* N contrast, we identified pathways enriched in gene targets of DEMs, the expression of which changes in the comparisons, and in DEG targets of DEMs, with only partially overlapping results. Some pathways, such as “Cell cycle”, “Purine metabolism” and “Pathways in cancer”, were found in both cases, whereas others, such as “P53 signaling” was identified by the first, less conservative strategy. Conversely, “Wnt signaling” and “Colorectal cancer” pathways were found to be specifically enriched in DEG targets of DEMs (Additional file 1: Table S4). It should be noted that “Pathways in cancer” is a collection of many pathways, including various cancer hallmarks such as EMT, and is an indication that many important processes may be under the control of identified miRNAs.

### Mixed regulatory circuits involving interplay of miRNAs and transcription factors contain many “cancer genes”

miRNAs regulate target genes mainly at the post-transcriptional level, operating in highly interconnected regulatory networks and pathways, with complex cross-talk of miRNAs and transcription factors (TFs), which are frequently master regulators of biological processes. miRNA expression can be activated or repressed by transcription factors (TFs), whereas mRNAs encoding TFs can be silenced by miRNAs. In many cases, key mixed regulatory circuits involve miRNAs, TFs and common target genes. Thus, miRNAs and TFs can form feedback or feedforward loops, cooperating to switch or tune gene expression.

To account for the interplay of miRNAs and TFs, we have recently developed a new method for the integrated analysis of target prediction, MAGIA2 [23], which allows one to dissect regulatory complexity by exploiting target predictions, in combination with information on experimentally validated TF-miRNA and TF-gene interactions, and which can identify both negative and positive expression profile correlations. By applying this method to the matched expression profiles of all genes and to the 70 DEMs in TN and/or MT comparisons, we were able to reconstruct mixed regulatory networks involving miRNAs and TFs. Our results show that 18 miRNAs are involved in the strongest 127 interactions identified as significant by MAGIA2 (Additional file 1: Figure S7A). Then, we focused on the identification of two types of triangular mixed circuits representing putative feed-forward or feed-back loops with interplay between transcriptional and post-transcriptional regulation: (i) circuits in which one TF regulates both a given miRNA and its target gene, and (ii) circuits in which one miRNA regulates both a given TF and its regulated gene. Additional file 1: Figure S7B reports 20 significant mixed circuits involving five distinct miRNAs which interact with 6 TFs and 15 non-TF protein-coding genes.

## Discussion

A straightforward interpretation of the data published thus far is complicated by the relatively small overlap between results obtained with differing analytical platforms, sample cohorts and bioinformatics methods [24]. In this study, we carried out a genome-wide integrative analysis of miRNA and gene expression profiles in 77 CRC samples, including normal colon mucosa, primary tumor and liver metastasis, in order to identify miRNA-gene relationships significantly supported by expression data and involving differentially expressed miRNAs, with the ultimate aim of discovering regulatory circuits and miRNA-affected cellular pathways specifically associated with tumor progression.

As shown in breast cancer by Farazi et al. [25], we observed that both miRNA and gene expression profiles efficiently separate tumor from normal samples. However, miRNAs proved to be more informative than genes in distinguishing primary colorectal tumors from liver metastases. Interestingly, gene expression profiles from tumor and metastatic samples obtained from the same patient tended to cluster together. Thus, based on its gene expression profile, a liver metastasis is more similar to the matched primary tumor than to the liver metastases of other patients, suggesting that metastasis development is a patient-specific process.

The large majority of miRNAs and genes with varied expression in the T *vs* N comparison remain stable after metastasis development (59 miRNAs out of 62, 95%; 424 genes out of 455, 93%). Three-quarters of miRNAs modulated in the M *vs* T comparison are invariant in the T *vs* N comparison (9 out of 12), whereas only 50% of genes are modulated in the M *vs* T comparison but not in the previous one.

DEMs between sample classes were identified, considering the whole set of samples and the smaller group of per-patient matched samples in parallel. The six- fold lower number of DEMs observed in the M *vs* T contrast, compared with T *vs* N (and M *vs* N) contrasts, indirectly indicates the similarity between tumor and metastatic tissues. This is particularly remarkable also considering that, as discussed for miR-122, a normal liver contribution to M transcriptome cannot be completely ruled out.

It has been hypothesized that miRNA down-regulation fosters invasive and metastatic behavior of cancer cells, since overall reduction of miRNA expression levels has been reported as a general trait of human cancers [26], and repression of miRNA biogenesis in cancer cell lines promotes cell proliferation and invasion [27]. In contrast, Volinia et al. [28] showed that the most common event in solid tumors is gain in miRNA expression, whereas Farazi et al. [25], comparing normal and cancer tissues, did not detect any change in total miRNA content. We did not observe any prevalence of up- or down-regulation of miRNAs in our tumor samples, either when considering the numbers of up- and down-regulated miRNAs (29 and 33, respectively, in the T *vs* N contrast) or when comparing the distributions of absolute expression values of DEMs in the same contrast.

miRNAs differentially expressed between tumor and normal mucosa include ones previously described as members of a “signature” common to various types of solid tumors [1]. Many of them have also been implicated in the molecular and biological processes which drive tumorigenesis in CRC (Additional file 1: Tables S1 and S2). Relevant examples are miR-143, miR-145, miR-125b and miR-21 (associated with cell growth and survival), the miR-17-92 cluster, miR-20 and miR-100 (involved in uncontrolled cellular proliferation), the miR-183 cluster and miR-31 (implicated in cell migration), and miR-150 (potential biomarker of prognosis and therapeutic outcome in CRC). Interestingly, miR-139-5p, the most down-regulated in the T *vs* N comparison, has very recently been identified as a member of a signature predictive of the clinical aggressiveness of stage II CRC [29]; in addition, miR-224, the most up-regulated together with miR-183 in the same comparison, has been identified for its ability to distinguish CRC by means of proficient or deficient DNA mismatch repair machinery [30]. For some of these miRNAs, the tumor-promoting or -suppressing functions in both CRC and other tumors have already been suggested. However, considering the large number of mRNAs regulated by each miRNA, it is very likely that two or more genes from different molecular pathways may be altered in their expression and, considering the tissue specificity of miRNA activity, strict classification of cancer-associated miRNAs into onco- or tumor-suppressor miRNAs may be an over- simplification. A clear-cut example is miR-10b: the expression of this miRNA has been correlated with migration and invasion in esophageal cancer cell lines and in breast cancer patients, thus suggesting its tumor-promoting role [31,32]. However, different results have recently been reported in gastric cancer, in which the silencing of miRNA-10b by methylation was associated with an increase in tumor cell growth through the activation of the oncogene MAPRE (microtubulus-associated protein RP/EB family, member 1) [33]. Matching this report, but in contrast with others [34], in our dataset miR-10b was down-regulated not only in the T *vs* N comparison but also in that of M *vs* T. miR-10b is one of the miRNAs known to be which is important in the EMT transition, and is involved in cell cycle regulation as well as in cancer recognition by the immune system [35-37]. It has several predicted targets according to the reconstructed networks, including two genes encoding proteins involved in ribosomal RNA biogenesis: UTP14 encodes the U3 small ribonucleoprotein homolog. DKC1 encodes Dyskerin, an essential nucleolar protein involved in cell proliferation, where it is required for the pseudo-uridylation of ribosomal RNA molecules, and for stabilization of the telomerase RNA component. Dyskerin overexpression has been recognized as a negative prognostic factor in advanced stage hepatocellular carcinomas [38].

Although purely exploratory in nature, due to the relatively low number of subjects analysed, our data support the prognostic value of miR-10b (Additional file 1: Figure S6), in line with available evidence regarding the role played by this microRNA in cancer biology, according to both preclinical [32,34,39-41] and clinical models [34,41-43]. miR-10b over-expression has been associated not only with enhanced aggressiveness of malignant cells in a variety of experimental models [32,34,39-41,44,45], but also with worse prognosis in patients with breast [34,43] and pancreatic carcinoma [41,42]. To our knowledge, our results suggest for the first time the potential involvement of miR-10b in CRC, with special regard to the modulation of the biological behavior of metastatic disease, and deserve further investigation.

When T and M samples were compared in unpaired analyses, only 5 over- and 6 under-expressed miRNAs were obtained. Two miRNA pairs were characterized by inverse down- modulation in the tumor toward metastasis transition: miR-100 and miR-99a according to the unpaired comparison.

The similarity in miRNA expression in later stages of tumor progression may reflect the need to maintain the tumor-specific processes required for tumorigenesis and cancer progression. However, it should be emphasized that any malignant tumor is made up of a heterogeneous cell population and that the differences measured in gene profiling experiments are thus the result of average changes occurring within the tumor.

The integrated analysis method has proved to be very useful in previous studies with relatively limited numbers of samples, but was expected to be more powerful when applied to large matched miRNA-gene expression datasets, as in this study (i.e., with limited dimensionality curse, imbalance between the number of estimated genes, and hybridizations to different samples). In fact, after controlling for multiple testing and using a stringent significance criterion (FDR < 0.01), we were able to identify a set of 3,078 trustworthy miRNA-target relations involving 117 (39%) of 309 selected miRNAs. We then defined a putative post-transcriptional regulatory network in the light of the information regarding differentially expressed miRNAs and genes in the T *vs* N and M *vs* T comparisons. The T *vs* N network includes two components (unconnected sub-networks) involving respectively 6 up-regulated and 17 down- regulated miRNAs together with their putative target genes, some of which are significantly differentially expressed in the same contrast. The biological meaning of the smaller component (Figure 2A), pertaining to the 6 miRNAs up-modulated in the T *vs* N contrast, is evidenced by the large proportion of significantly modulated genes among the set of predicted target genes represented in the network. This observation emphasizes the fact that the pure number of up- or down-regulated miRNAs may not really be important in predicting the effect of miRNA regulation on cell behavior, for which gene expression is a proxy. Some genes are shared predicted targets of different miRNAs: the PDCD4 gene, a tumor suppressor gene, appears to be the target of miR-21, miR-182 and miR-183, all up- regulated in the T vs N comparison.

The interplay between the sub-networks suggested to be modulated by miR-21 and miR-182 deserves comment. miR-21 is an oncomiR whose role in “licensing” and supporting the neoplastic process from the earliest step of tumorigenesis is well-known in several types of solid tumors; its over-expression has in fact been detected in pre-neoplastic lesions of colon mucosa and in advanced adenocarcinomas [46]. The connection between miR-21 and miR-182 is particularly intriguing, in the light of the role of miR-182 in cytoskeleton reorganization, a process which favors the epithelial to mesenchymal transition and fosters cell proliferation and invasion. Among the predicted miR-182 targets, ENTPD5 was differentially down-regulated in our analysis. The gene product is a member of the family of ectonucleoside triphosphate diphosphohydrolase (E-NTPDases) enzymes which hydrolyse extracellular tri- and diphosphonucleosides, are components of cellular purinergic signaling, and are involved in energy metabolism [47]. Mikula et al. recently showed that both ENTPD5 mRNA and protein levels progressively decrease during the transition from normal colon mucosa, through adenoma to adenocarcinoma [21]. This finding is in line with our results, which also indicate miR-182 as a possible regulator of ENTPD5 expression.

The post-transcriptional regulatory network with miRNAs differentially expressed in the comparison of M *vs* T was smaller: only 5 DEMs were modulated, each defining a network component (Additional file 1: Figure S4). The substantial overlap of miRNAs observed to be differentially expressed in T *vs* N and M *vs* T comparisons, the absence of differentially expressed genes in the MT network, together with the paucity of pathways significantly modulated in the same comparison (involving supported miRNA target genes) suggest a limited role of miRNAs in metastasis development, or in alternative a regulatory influence operating mainly at the translational level. This result again stresses the strict dependency of malignant cells on early molecular events acquired during tumorigenesis. In this respect, we observed that 22 miRNAs involved in EMT varied during tumor progression: 19 were differentially expressed in primary tumors compared with normal tissue and one in liver metastasis compared with primary tumor, and one, miR-10b, was common to both comparisons, as shown in Additional file 1: Figure S5. We observed that the expression of many DEMs involved in EMT was modulated in tumor development (T *vs* N comparison) and then remained stable or at similar levels in metastasis. We identified various KEGG pathways modulated in the T *vs* N contrasts by examining the expression profiles of all genes supported as targets of DEMs. The implementation of a method based on gene sets allowed us to identify significantly modulated pathways, rather than simply enriched in genes representing the target of specific miRNA groups. For instance, focusing on T *vs* N up-regulated DEMs, miR-182 is involved together with miR-21, miR-18a, miR-1246 and miR-183 in the modulation of cancer-related pathways, and with miR-150 and miR-183 in the reprogramming of energy metabolism (purine and selenoaminoacid metabolism), in which various down-modulated DEGs were found, including ENTPD5 (Figure 2 and Additional file 1: Table S4). At the same time, miR-182 was grouped in the cell cycle pathway together with down-regulated miRNAs. In this way, it may directly regulate CDKN2B (a CDK inhibitor controlling G1 progression, down-modulated) and collaborate with down- regulated miRNAs such as miR-145 and miR-195, in modulating key genes such as CDC25B, MYC and PRKDC, involved in cell cycle progression checkpoints and DNA damage response (Figure 3 and Additional file 1: Table S4).

Furthermore, we reconstructed mixed regulatory networks and specific circuits involving miRNAs, TFs and common target genes. Among some of the most significant interactions in the mixed network associated with the strongest correlations, we found the oncogenes MEIS1 and MYC, RBMS3 (encoding an RNA binding protein of the c-myc family), SVEP1 (involved in cell adhesion), LPP (of the LIM family of proteins involved in cell adhesion and motility), CASP7 (a caspase important in the execution phase of cell apoptosis) and the validated relation miR-145/FLI1, involving a well-known “cancer gene”.

These circuits show some of the top interactions involving miRNAs, TFs and common target genes. An interesting network component includes miR-145, the TF MEIS1 (a development and neoplasia gene) and the REV3L gene (the catalytic subunit of DNA polymerase zeta, involved in DNA repair and genome stability), all down-regulated in NTM progression. It is known that inhibition of REV3 expression induces persistent DNA damage and growth arrest in cancer cells [48].

Connected circuits involve SOX9, an important cancer gene, a TF which antagonizes β-catenin, inhibits TCF activity in cancer cells and modulates cell proliferation [49]. The other component comprises several circuits involving miR-17, miR-195 and miR-497 together with NF1 (a hypoxia-activated gene), TFAP4 (another cancer gene with prognostic importance in gastric carcinoma), MYC and HNF1A, and common target genes, which can mainly be classified as cancer genes.

## Conclusions

Using a large dataset of matched miRNA and gene expression profiles in normal mucosa, primary cancer and metastasis, we describe the interplay of modulated miRNA groups in the regulation of gene expression, which in turn affects modulated pathways important for tumor development. The suppressor activity of miR-182 on the ENTPD5 gene was identified for the first time and confirmed in an independent set of samples.

## Methods

### Patients and collection of tissue samples

For this study, 46 patients with sporadic colorectal adenocarcinomas, who underwent surgery at the University of Padova (Surgery Unit, Department of Surgery, Oncology and Gastroenterology) between March 1994 and September 2008, were selected from the institutional CRC database. Patients with a known history of a hereditary colorectal cancer syndrome were excluded. The Ethics Committee of the University Hospital of Padova approved the study. All patients provided written informed consent. Enrolled patients did not receive any neo-adjuvant treatment. Normal mucosa samples were taken at a minimum distance of 10 centimeters from the tumor site. All samples were immediately snap-frozen in liquid nitrogen and stored at −80° until use.

### RNA extraction

7 μm sections from each tissue sample were prepared using a Leica CM 1950 cryostat (Leica Microsystems, Wetzlar, Germany). Hematoxylin and eosin stained sections of each specimen were prepared and re-evaluated by one experienced pathologist (G.E.); only samples with more than 80% of vital tumor tissue were considered for RNA extraction *in toto*. Laser microdissection was performed on a few frozen samples of primary tumours and metastases with a proportion of neoplastic cells lower than 80% using LMD-6000 Laser Microdissection System (Leica Microsystems, Wetzlar, Germany). Total RNA from samples was isolated using Trizol (Life Technology Corp, Carlsbad, CA, USA) according to the manufacturer’s instructions. RNA concentration was quantified on a NanoDrop 1000 Spectrophotometer (NanoDrop Technologies, Waltham, MA, USA). RNA quality was evaluated by RNA 6000 Nano LabChip (Agilent Technologies, Santa Clara, CA, USA) on an Agilent 2100 Bioanalyzer. Samples with RNA integrity number (RIN) < 6 were excluded.

### Expression profiling

miRNA microarray hybridization was performed from total RNA with the Affymetrix GeneChip miRNA Array 2.0 (Affymetrix Santa Clara, CA, USA). Briefly, 100 ng of total RNA from each sample were labeled with the FlashTag Biotin RNA Labeling Kit (Genisphere, Hatfield, PA, USA) and samples were then hybridized according to the manufacturer’s instructions.

GeneChip Human Exon 1.0 ST (Affymetrix) microarray hybridization was performed from the same total RNA extracted for miRNA profiling. Total RNA (100 ng from each sample) was labeled with the Ambion WT expression kit (Ambion Inc, Austin, TX, USA), as provided by the manufacturer. End-labeling, hybridization, washing and scanning were performed according to the GeneChip Whole Transcript (WT) Sense Target Labeling Assay user manual (Affymetrix), and scanned with an Affymetrix GCS 3000 7G scanner.

For both miRNAs and exon arrays, a first quality control check was performed with Affymetrix® Expression Console™ software (v.1.0) to determine the success of hybridizations. miRNA and gene expression measures were reconstructed from .cel files by using the Robust Multichip Average (RMA) method. Signals of 41 probes per gene, on average, were summarized to estimate gene expression with EntrezGene-based custom CDF (http://brainarray.mbni.med.umich.edu/Brainarray), obtaining expression profiles of 22,517 genes.

Detailed quality control of samples was carried out with R software: Normalized Unscaled Standard Error (NUSE) and Relative Log Expression (RLE) for global quality of signals in each array assessment, and MA plots before and after RMA for identification of biases associated with specific intensity classes.

miRNAs detected in fewer than 20 samples were discarded, without filtering out miRNAs undetected only in one sample class.

### miRNA and gene differential expression

A Significance Analysis of Microarrays (SAM) with an unpaired two-class design was performed to identify differences in miRNA expression between groups of normal mucosa (N), primary tumor (T) and liver metastases (M) samples.

Differentially expressed genes (DEG) between groups of N, T and M samples were identified with SAM with an unpaired two-class unpaired design. The cut-off for significance (determined by tuning parameter delta) corresponded to a false discovery rate (FDR) < 0.01.

### Integrated analysis of miRNAs and gene expression profiles

We integrated target predictions with correlation-based miRNA and gene expression profile, to identify those regulatory relationships significantly supported by expression data. This analysis is based on the assumption that, at least for miRNAs acting on mRNA stability, the expression profile of a given miRNA is expected to be inversely correlated with those of its true targets. First, miRNA target predictions were obtained with miRanda-miRSVR, a method able to identify miRNA-mRNA interactions involving the seed sequence as well as non-conserved and non-canonical sites [50].

Using a genome-wide approach, we considered over four million miRNA-gene relationships predicted with a “good” score, 25% of which involved evolutionarily conserved target sites. Pair-wise Pearson correlations between miRNA and predicted target gene expression profiles were then calculated and assigned a statistical significance (p-value). The FDR (q-value) was used to correct the correlation significance for multiple testing. Only miRNA-target relationships with significant correlations (FDR < 0.01) were considered to be supported by expression profiles.

Considering that expression values ranged from 0 to 15 in log2 scale and that 70% of miRNAs were weakly expressed, we then focused only on miRNAs with signal over 5, arbitrarily set to the average value of miRNA expression.

### Enrichment of KEGG pathways

To identify significantly perturbed KEGG pathways for each considered comparison, we applied the statistical procedure GAGE [51], which takes into account gene expression variations in both directions, to genes turning out to be supported targets of DEMs (*p*-value < 0.05). In particular, two strategies were implemented to identify pathways enriched in genes targets of DEMs, the expression of which changes in the considered comparisons, and pathways enriched in DEGs targets of DEMs.

### Survival analysis

We investigated the association between the expression levels of 26 DEMs (present in reconstructed post-transcriptional regulatory networks) in biopsies obtained from distinct primary [n = 26] or metastatic [n = 20] colorectal cancers and patients’ disease- specific survival (interval between diagnosis of primary or metastatic disease and death by disease or last follow-up).

Given the relatively low sample size, only univariate survival analysis was performed, and the Cox proportional hazard regression model was used, assuming a linear functional form of the covariates being assumed. The risk associated with a unit increase in miRNA levels was expressed as hazard ratio (HR) and its 95% confidence interval (CI). With Bonferroni’s p-value adjustment for multiple comparisons, the alpha level of significance was set at 0.002. In order to illustrate prognosis associated with different levels of the relevant miRNAs, Kaplan-Meier survival curves were generated after dichotomizing (high *vs* low categories) originally continuous covariates based on the median values of miRNA expression levels. All analyses were performed with Stata/SE software (version 11.0, StataCorp LP, College Station, TX, USA).

### Quantitative RT-PCR

To confirm array data for miR-150, miR-146a, miR-10b, miR-122 and miR-210 and to validate miR-145/c-Myc and miR-182/ENTPD5 relationships, we conducted qRT- PCR experiments, as previously described [52]. Briefly, experiments were performed three times in triplicate with a LightCycler 480 II (Roche Diagnostics, Basel, Switzerland) and were analysed by the ∆∆Ct method. Quantification of the five selected miRNAs, miR-145 and c-Myc was normalized against internal housekeeping controls, selected for their minimum variability (measured as expression profile Shannon entropy). RNU44 and GAPDH were used as internal controls, respectively for miR-182 and ENTPD5 gene expression quantification.

### Luciferase reporter assay

HEK293T cells were plated in 24-well plates at 8×104 cells/well and cotransfected using Lipofectamine 2000 (Invitrogen), with 500 ng pMir target vector customized for ENTPD5 3′UTR (OriGene Technologies, Rockville, MD, USA) and 250 ng pRL-TK (Promega, Milan, Italy) following the manufacturers’ instructions. For miR-182 analysis, cells were cotransfected with non-target RNA (Tema ricerca, Bologna, Italy) as negative control or miCENTURY OX miNatural for hsa miR-182 (Tema ricerca), in triplicate. For the above analyses, cell lysates were analysed 30 hours after transfection by the Dual-Glo Luciferase Assay System (Promega) and experiments were independently repeated three times.

### Reconstruction of mixed miRNA-TF networks and circuits

Matched miRNA and gene expression data were analysed with MAGIA2 to construct mixed miRNA-TF networks and circuits. MAGIA2 was run on the expression data for the top 75% of genes with greatest differences in expression and of 70 DEMs in TN and/or MT comparisons. The TargetScan (default settings) method was selected for miRNA target prediction. For TFs, MAGIA2 utilizes experimentally validated TF– miRNA interactions from mirGen2.0 and TransmiR and on TF–gene interactions from the ‘TFBS conserved’ track of the UCSC human genome annotation. Pearson’s correlation was used as a profile association measure.

## Abbreviations

miRNA: microRNA; CRC: Colorectal cancer; N: Normal colon mucosa; T: Primary colon tumor; M: Liver metastasis; DEM: Differentially expressed miRNA; DEG: Differentially expressed gene; GEO: Gene expression omnibus; qRT-PCR: Quantitative real time PCR.

## Competing interests

The authors declare that they have no competing interests.

## Authors’ contributions

SP, AB, MB and SB carried out expression data analysis, regulatory network reconstruction and pathway enrichment analysis, and drafted the manuscript; SM, AF and LP performed laboratory experiments; ER carried out microarray hybridization, GE performed histopathological re-evaluation of tissues; MR made histopathological evaluations of tissues after surgery; PLP, SM and DN performed the surgery and collected patients’ histopathological data; SM carried out survival analysis and helped to draft the manuscript; DN participated in the design and coordination of the study; SB and PZ conceived and coordinated the study and drafted the manuscript. All authors read and approved the final manuscript.

## Supplementary Material

Additional file 1 **The following additional data is available with the online version of this paper.** Additional data includes Supplementary Methods and Results, Additional file 1: Figures S1- S7 and Additional file 1: Tables S1-S4. Click here for file
