# Supplementary material for: Impact of microRNAs on regulatory networks and pathways in human colorectal carcinogenesis and development of metastasis
Source: BMC Genomics. 2013 Aug 29;14:589. doi: 10.1186/1471-2164-14-589 (PMC3766699; doi:10.1186/1471-2164-14-589)
Supplement: Additional file 1 — The following additional data is available with the online version of this paper. Additional data includes Supplementary Methods and Results, Additional file 1: Figures S1- S7 and Additional file 1: Tables S1-S4. [file 1471-2164-14-589-S1.pdf]

Impact of microRNAs on regulatory networks and pathways in human colorectal carcinogenesis and metastasis development.

Pizzini, Bisognin et al.

Additional data

In this document are included: Supplementary Methods and Results; Supplementary Figure S1; Supplementary Figure S2, Supplementary Figure S3, Supplementary Figure S4, Supplementary Figure S5 and Supplementary Figure S6; Supplementary Figure S7; Supplementary table S1; Supplementary table S2; Supplementary table S3 and Supplementary table S4.

Supplementary Results and Methods

Results

Differentially expressed miRNAs

We identified differentially expressed miRNAs by paired comparisons carried out on subsets of samples matched by patient (f.i. the subset of T and N samples including only sample pairs coming from the same patient was considered for the paired T *vs* N comparison). We found 34, 38 and 5 DEMs, respectively in T *vs* N, M *vs* N and M *vs* T comparisons (Table S2). As shown in Figure S2 (top panels), most DEMs identified with the paired test were confirmed with the unpaired test conducted on the larger dataset. The figure shows, for each comparison, the numbers of DEMs obtained with paired and unpaired designs, and intersections thereof. Five miRNAs vary between primary tumor and liver metastasis obtained from the same patient, and only miR-100, up-regulated in metastasis, is differentially expressed only in the M *vs* T comparison.

When T and M samples were compared in paired analyses, only 5 over-expressed miRNAs were found. Two miRNA pairs were characterized by inverse down-modulation in the tumor toward metastasis transition: miR-139-5p and miR-150. Common to paired and unpaired comparisons were miR-210 (whose over-expression in solid tumors has been associated with hypoxia, cell survival and invasion) and miR-100 (which is considered a regulator of the ATM/mTOR pathway).

Methods

Differentially expressed miRNA in pairwise group comparisons, involving groups of samples matched per patient (e.g., T *vs* N samples matched per patient) were calculated with SAM using a two-class pairsed design. The cut-off for significance (determined by tuning parameter delta) corresponded to a false discovery rate (FDR) < 0.01.

Supplementary Figures

Supplementary Figure S1 Samples classification and heatmap based on 309 miRNAs (top panel) and 15,761 genes (bottom panel) expression profiles. In both panels, color-coding of samples reported in three different lines refers to different information. First line indicates tissue type (N, normal colon mucosa; T, primary tumor and M, liver metastasis) as shown in the legend. The two lines below indicate the per-patient matching of samples, separately for triples (upper line) and couples (lower line) of samples from the same patient (i.e. samples from the same patient are in the same color).

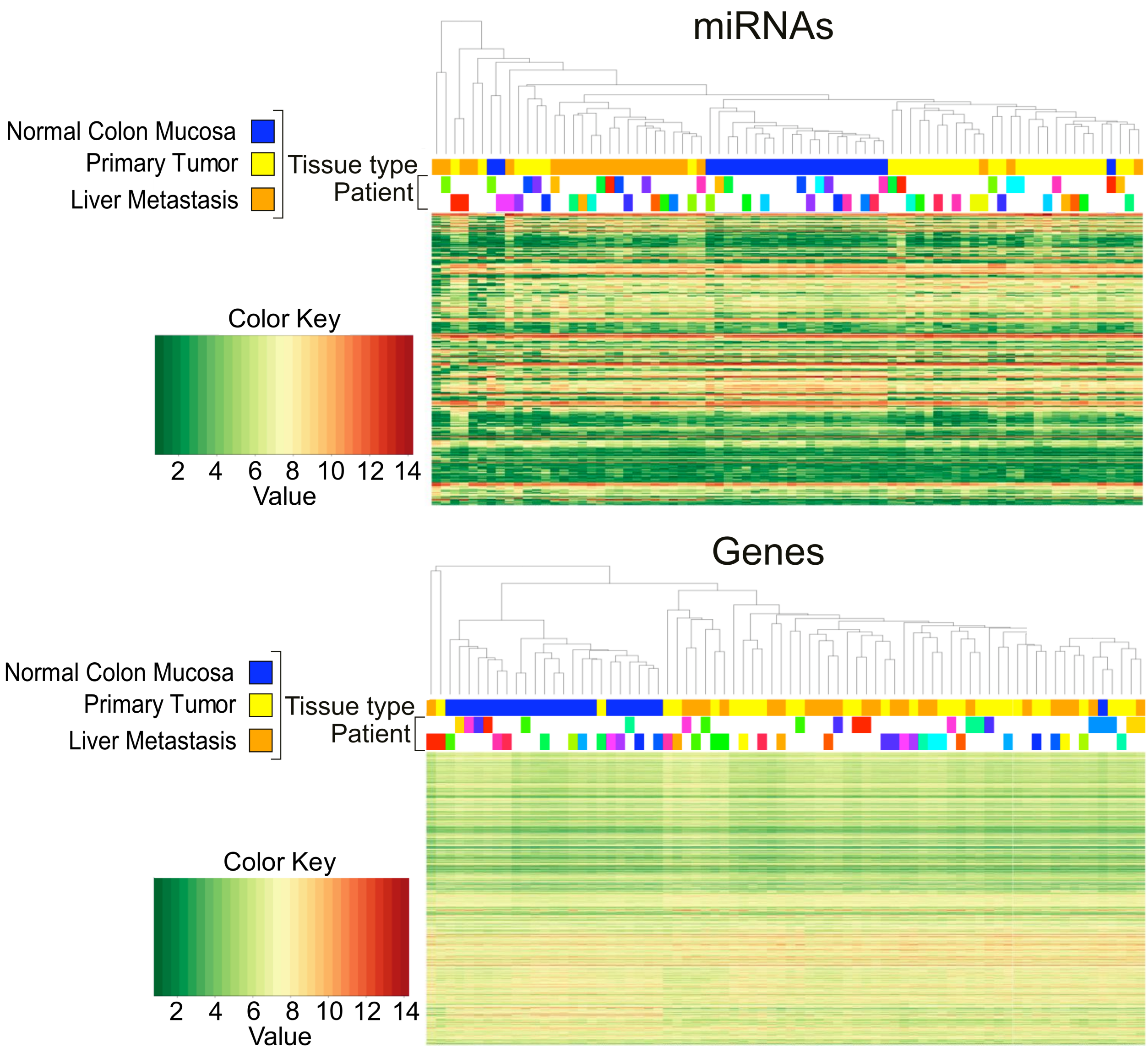

Supplementary Figure S2.Venn diagrams of intersections among DEMs obtained with different contrasts and methods. Top: overlap of DEMs found in the in same contrast (e.g., T *vs* N) with paired and unpaired tests; bottom: overlap among DEMs obtained by the same test applied to different contrasts.

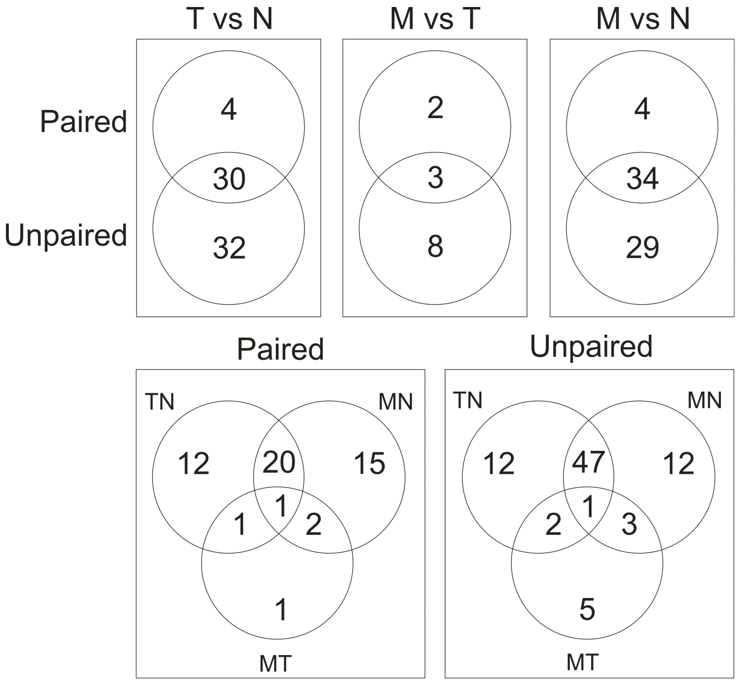

Supplementary Figure S3. Expression profiles in considered sample classes of 22 miRNAs reportedly involved in EMT (Epithelial to Mesenchimal Transition) that are differentially expressed in the T vs N and/or M vs T comparisons.

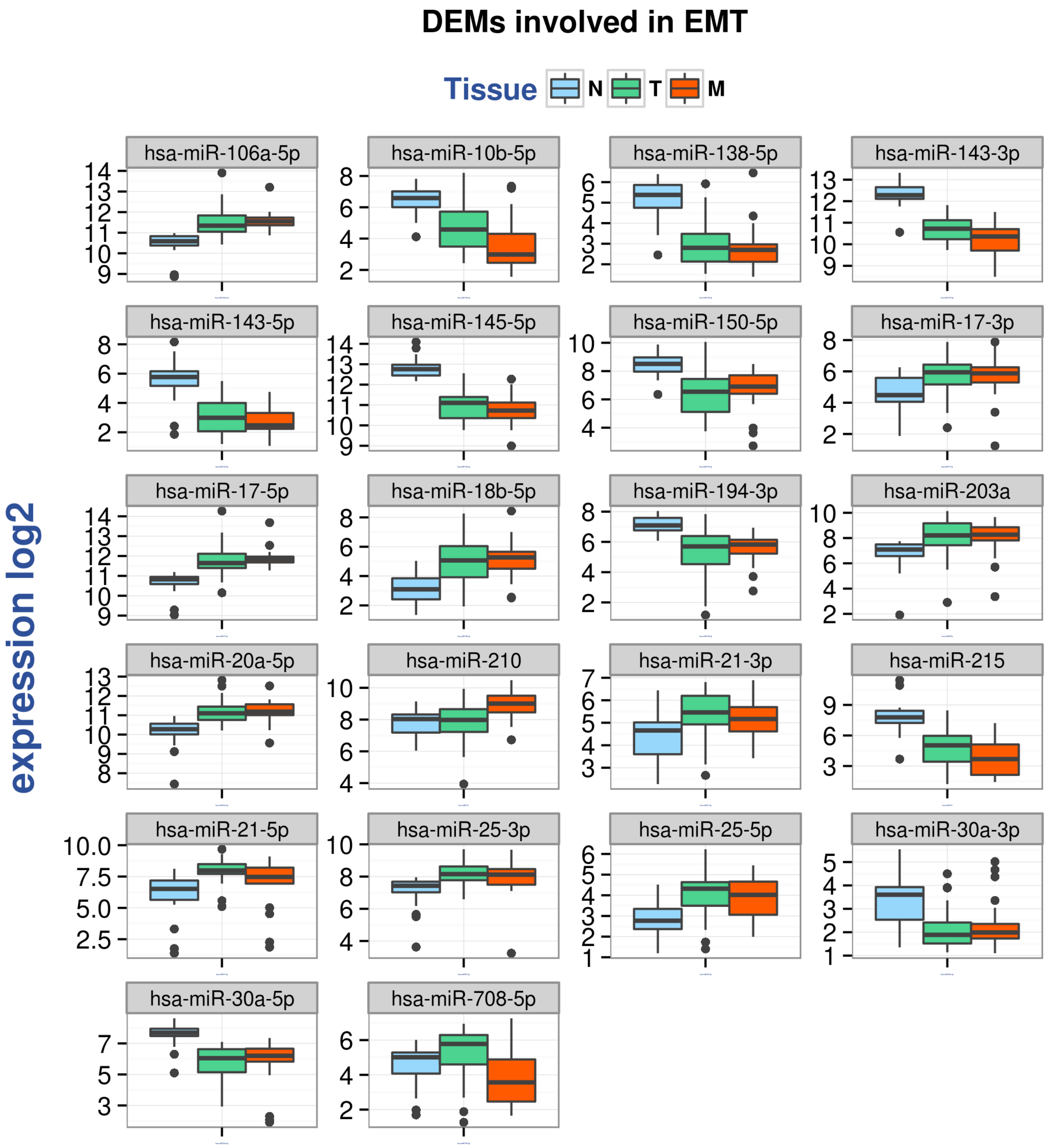

Supplementary Figure S4 Differential expression of miR-146a and miR-150 is concordant, independently by samples set and methodology. Upper panel shows, for each miRNA, Spearman rank correlations and associated p-values between miRNA expression estimations obtained with microarrays and qRT-PCR on the same set of 78 samples, including normal colon mucosa (N), primary tumor (T) and liver metastasis (M). Lower panel shows the variation of miR-146a and miR-150 in the three tissue types, measured in the set of 78 samples both by arrays and qRT-PCR, and by qRT-PCR on an independent set of 21 samples.

A

microRNA	Spearman	Spearman Pvalue
miR-122	0,61	4.03e-09
miR-10b	0,31	0,006
miR-150	0,68	7.61e-12
miR-146a	0,58	3.59e-08
miR-210	0,32	0,004

B

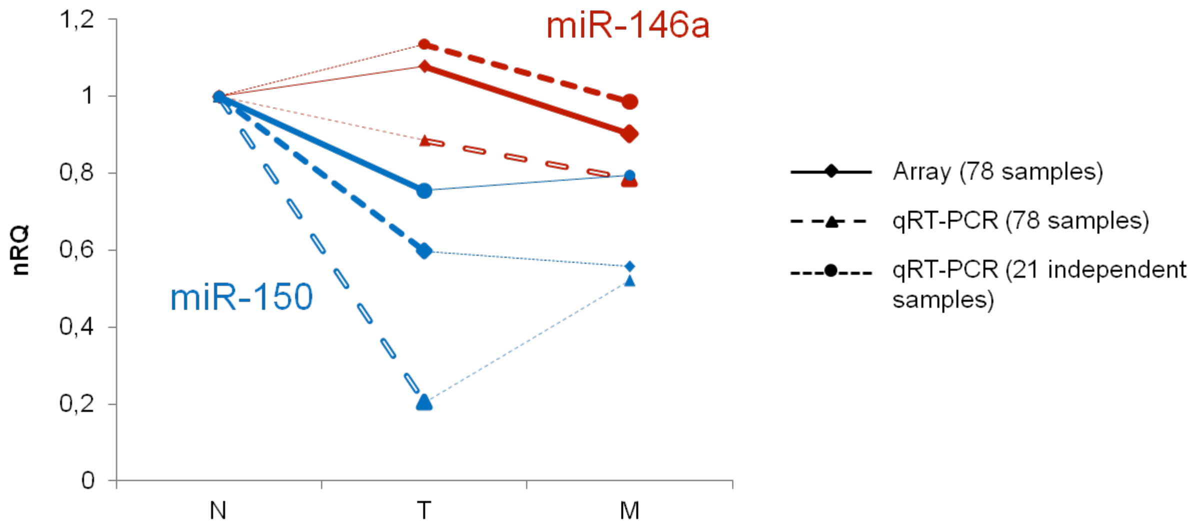

Supplementary Figure S5. Post-transcriptional regulatory network of miRNAs modulated in M *vs* T contrast. The bipartite network represents DEM up- and down-modulated (red and green triangles, respectively) in the M *vs* T comparison, supported target genes (circles) and their relations (gray dotted lines).

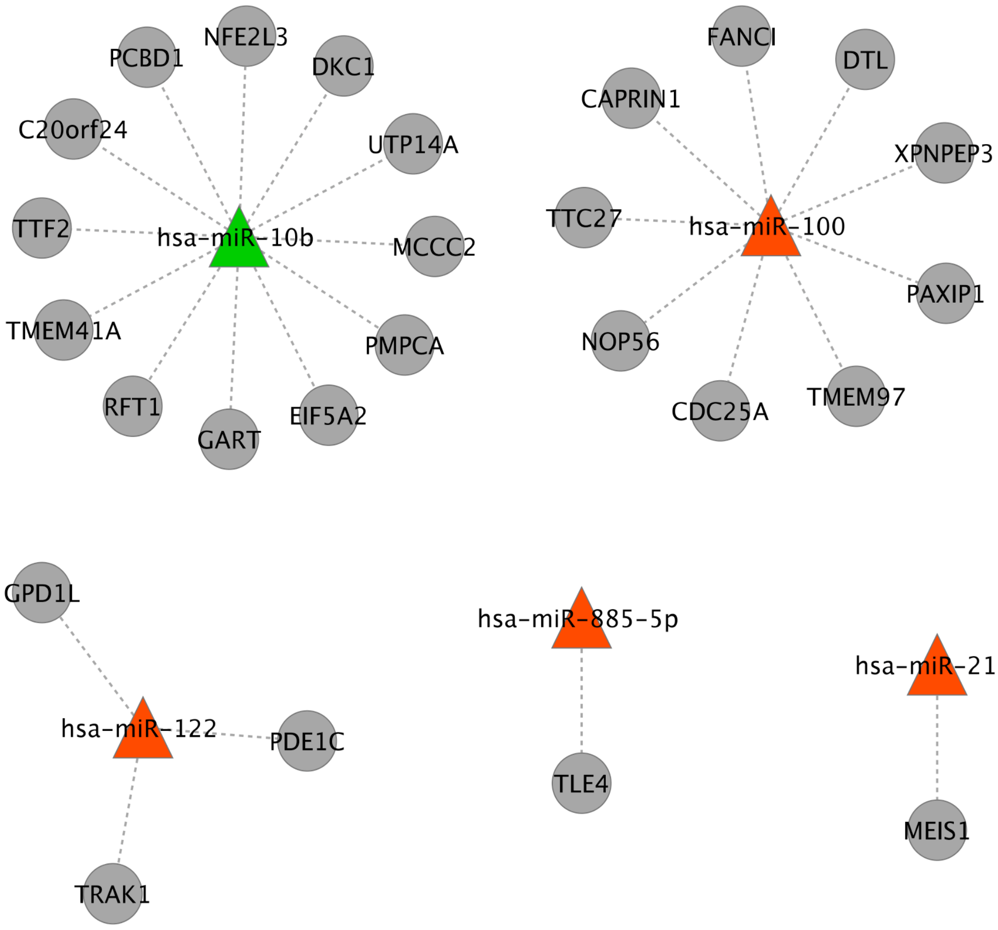

Supplementary Figure S6. Kaplan-Meier overall survival curve was plotted based on miR-10b expression showing relationship between miR-10b expression and survival in CRC cancer patients.

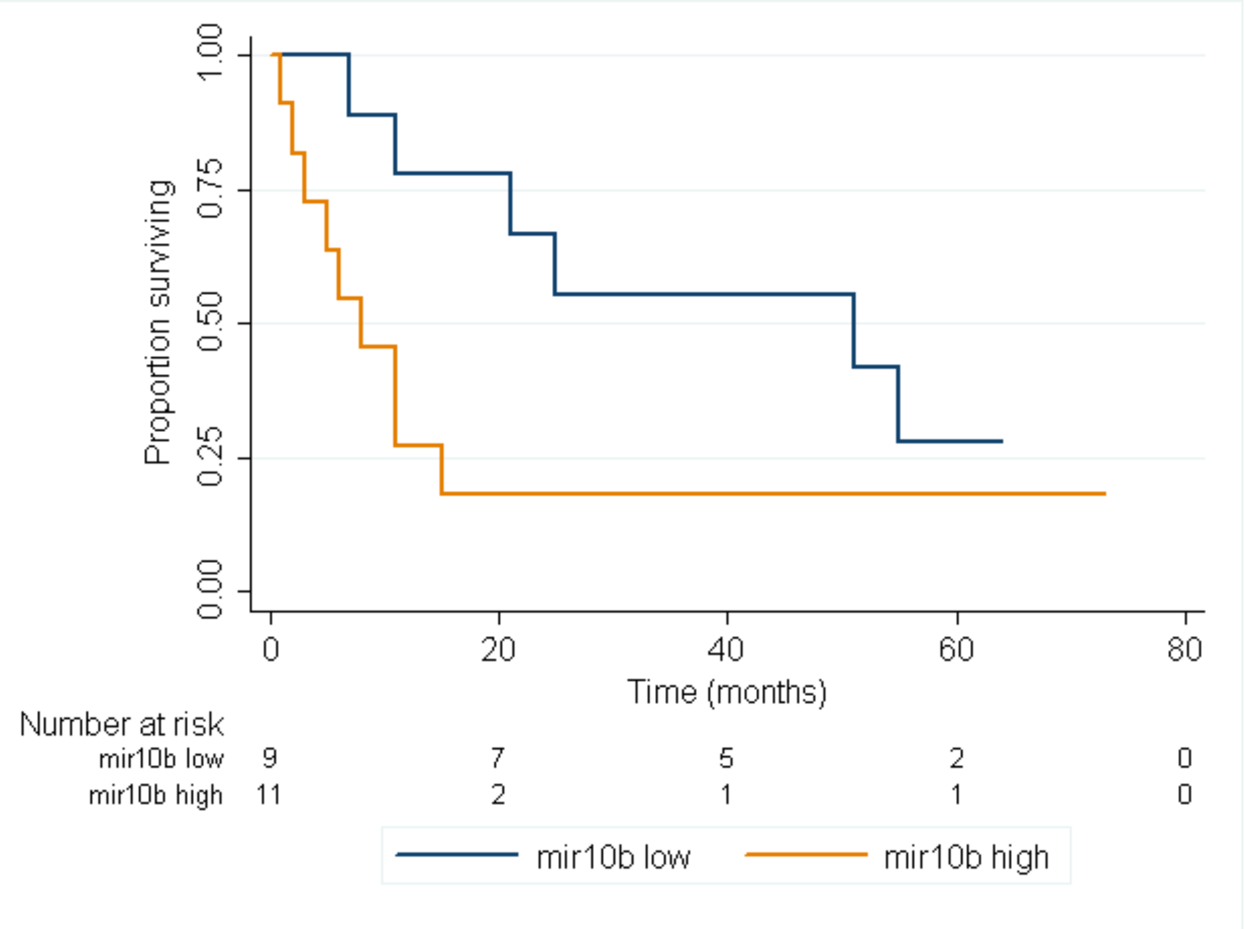

Supplementary Figure S7. Regulatory networks showing interplay among miRNAs and TFs. Networks were constructed using MAGIA[2], which takes into account positive and negative correlations between expression profiles of possibly interacting miRNAs, TFs and genes, according to predictions (see Methods). A. Regulatory networks showing the top 200 interactions between miRNAs, genes and TFs supported by expression data analysis. B. Top 20 mixed regulatory circuits involving TFs, miRNAs and common target genes, according to two circuit types: a TF that regulates both a given miRNA and its target gene, and a miRNA that regulates both a given TF and its regulated gene. In both panels, genes, TFs and miRNAs are shown as light blue circles, green circles, and orange triangles, respectively; positive and negative correlations are shown as red and blue arrows, respectively.

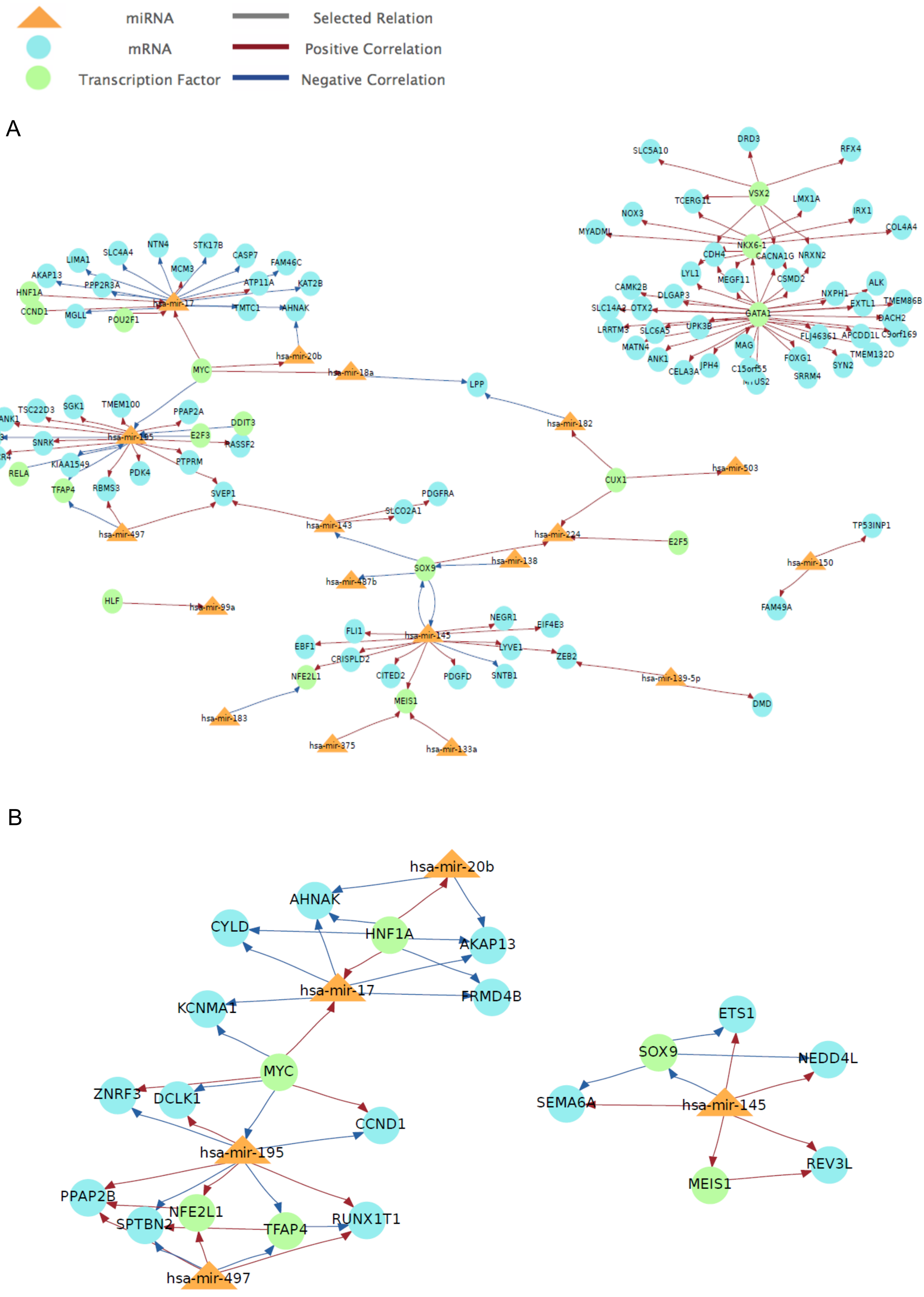
miRNA
mRNA
Transcription Factor
Selected Relation
Positive Correlation
Negative Correlation
A
B
hsa-mir-20b
AHNAK
CYLD
HNF1A
AKAP13
hsa-mir-17
FRMD4B
KCNMA1
MYC
ZNRF3
DCLK1
CCND1
hsa-mir-195
PPAP2B
NFE2L1
SPTBN2
TFAP4
RUNX1T1
hsa-mir-497
ETS1
SOX9
NEDD4L
SEMA6A
hsa-mir-145
REV3L
MEIS1

Supplementary Tables

Supplementary Table S1. miRNA differentially expressed in different contrasts, according to unpaired test.

T *vs* N DEMs		
Gene ID	Fold Change	q-value(%)
hsa-miR-139-5p	-3.272177914	0
hsa-miR-215	-2.988818352	0
hsa-miR-99a	-2.383513412	0
hsa-miR-143*	-2.366759526	0
hsa-miR-497	-2.34419323	0
hsa-miR-100	-2.324930718	0
hsa-miR-138	-2.238032916	0
hsa-miR-195	-2.107741275	0
hsa-miR-150	-2.069699018	0
hsa-miR-145	-1.91804478	0
hsa-miR-378	-1.850939299	0
hsa-miR-422a	-1.81814864	0
hsa-miR-375	-1.793533624	0
hsa-miR-30a	-1.793453612	0
hsa-miR-378*	-1.790182405	0
hsa-miR-133a	-1.773823053	0
hsa-miR-125b	-1.77084525	0
hsa-miR-10b	-1.713863042	0
hsa-miR-194*	-1.632313259	0
hsa-miR-143	-1.57945413	0
hsa-miR-342-5p	-1.526441766	0
hsa-miR-140-3p	-1.334735965	0
hsa-miR-28-3p	-1.299378565	0
hsa-miR-342-3p	-1.295561642	0
hsa-miR-149	-1.294815379	0
hsa-miR-574-3p	-1.251981975	0
hsa-miR-30a*	-1.206077017	0
hsa-miR-127-3p	-1.163578041	0
hsa-miR-130a	-1.116957708	0
hsa-miR-152	-1.116820485	0
hsa-miR-487b	-1.075980581	0
hsa-miR-379	-1.031769391	0
hsa-miR-125a-5p	-1.025384903	0
hsa-miR-421	1.000284994	0
hsa-miR-424*	1.003380509	0
hsa-miR-429	1.007465396	0
hsa-miR-21*	1.026996086	0
hsa-miR-106a	1.042630924	0
hsa-miR-17	1.091146105	0
hsa-miR-1290	1.093749518	0
hsa-miR-20a	1.111479331	0
hsa-miR-25	1.122075965	0
hsa-miR-17*	1.13475031	0
hsa-miR-25*	1.149337691	0
hsa-miR-552	1.208369576	0

hsa-miR-19a	1.221854517	0
hsa-miR-92a-1*	1.227938987	0
hsa-miR-181d	1.282578776	0
hsa-miR-29b-1*	1.328202727	0
hsa-miR-203	1.389987787	0
hsa-miR-886-5p	1.46179031	0
hsa-miR-20b	1.468807073	0
hsa-miR-1246	1.775916675	0
hsa-miR-1308	1.857166271	0
hsa-miR-31	1.877829229	0.509536597
hsa-miR-18b	1.909565741	0
hsa-miR-21	1.913670952	0
hsa-miR-18a	1.93394207	0
hsa-miR-503	1.950664843	0
hsa-miR-224	2.058520635	0
hsa-miR-182	2.187797216	0
hsa-miR-183	2.53733318	0
M *vs* N DEMs		
hsa-miR-215	-3.929340947	0
hsa-miR-497	-3.0947646	0
hsa-miR-195	-3.03721844	0
hsa-miR-10b	-2.817199413	0
hsa-miR-143*	-2.74101808	0
hsa-miR-139-5p	-2.438803085	0
hsa-miR-138	-2.395860799	0
hsa-miR-422a	-2.32174352	0
hsa-miR-375	-2.279264002	0
hsa-miR-145	-2.086918938	0
hsa-miR-133a	-2.078677786	0
hsa-miR-143	-2.067109714	0
hsa-miR-378*	-2.012477446	0
hsa-miR-378	-2.005904658	0
hsa-miR-30a	-1.758636411	0
hsa-miR-150	-1.741173237	0
hsa-miR-130a	-1.526273828	0
hsa-miR-194*	-1.510021172	0
hsa-miR-28-3p	-1.440290149	0
hsa-miR-140-3p	-1.365613614	0
hsa-miR-342-5p	-1.317922801	0
hsa-miR-27b	-1.166394486	0
hsa-miR-28-5p	-1.134539748	0
hsa-miR-192*	-1.122937275	0
hsa-miR-574-3p	-1.101312861	0
hsa-miR-30e	-1.067718574	0
hsa-miR-29c	-1.059051697	0
hsa-miR-342-3p	-1.058066906	0
hsa-miR-147b	-1.050725923	0
hsa-miR-199b-3p	-1.021067375	0
hsa-miR-152	-1.020728547	0
hsa-miR-30a*	-1.010530732	0
hsa-miR-25*	1.002975554	0
hsa-miR-17*	1.028867701	0.550533795
hsa-miR-106b*	1.071120871	0
hsa-miR-18a*	1.072760582	0

hsa-miR-20a	1.075735491	0
hsa-miR-92a-1*	1.094289701	0
hsa-miR-188-5p	1.098647694	0
hsa-miR-19a	1.106163101	0
hsa-miR-210	1.111334098	0
hsa-miR-20b	1.117501533	0
hsa-miR-106a	1.132522457	0
hsa-miR-17	1.236828699	0
hsa-miR-1275	1.250592958	0
hsa-miR-203	1.261763799	0
hsa-miR-483-5p	1.359232784	0
hsa-miR-552	1.391223984	0
hsa-miR-421	1.42187077	0
hsa-miR-886-5p	1.463191451	0
hsa-miR-1308	1.593441466	0
hsa-miR-885-5p	1.625067821	0
hsa-miR-181d	1.657513172	0
hsa-miR-424*	1.689271681	0
hsa-miR-224	1.747108666	0
hsa-miR-1290	1.998961285	0
hsa-miR-18b	2.026686411	0
hsa-miR-18a	2.065786605	0
hsa-miR-1246	2.384683531	0
hsa-miR-182	2.388952962	0
hsa-miR-503	2.616172225	0
hsa-miR-183	2.693341196	0
hsa-miR-122	8.312462259	0
M *vs* T DEMs		
hsa-miR-146a	-1.379160258	0
hsa-miR-708	-1.292819562	0
hsa-miR-15a	-1.212038005	0
hsa-miR-196a	-1.166896704	0
hsa-miR-10b	-1.103336371	0
hsa-miR-15b	-1.068075875	0
hsa-miR-210	1.114739685	0
hsa-miR-99a	1.483966648	0
hsa-miR-885-5p	1.506612383	0
hsa-miR-100	1.753641519	0
hsa-miR-122	7.943847008	0

Supplementary Table S2. miRNA differentially expressed in different contrasts, according to paired test.

T *vs* N DEMs		
Gene ID	Fold Change	q-value(%)
hsa-miR-139-5p	-3.843098125	0
hsa-miR-497	-3.09369775	0
hsa-miR-138	-2.7540045	0
hsa-miR-150	-2.743002375	0
hsa-miR-195	-2.73133675	0
hsa-miR-375	-2.59429075	0
hsa-miR-99a	-2.577299125	0
hsa-miR-133a	-2.322061088	0
hsa-miR-30a	-2.217122625	0
hsa-miR-145	-2.09149625	0
hsa-miR-342-5p	-1.919002875	0
hsa-miR-378	-1.917125125	0
hsa-miR-194*	-1.8245275	0
hsa-miR-143	-1.70583375	0
hsa-miR-140-3p	-1.665123125	0
hsa-miR-342-3p	-1.5621085	0
hsa-miR-30a*	-1.55852025	0
hsa-miR-422a	-1.505709875	0
hsa-miR-768-3p	-1.450829	0
hsa-miR-28-3p	-1.342629625	0
hsa-miR-574-3p	-1.284033875	0
hsa-miR-381	-1.1066095	0
hsa-miR-768-5p	-1.095674125	0
hsa-miR-20a	1.115748875	0
hsa-miR-106a	1.20225525	0
hsa-miR-17	1.29234875	0
hsa-miR-20b	1.41034325	0
hsa-miR-106b*	1.460387125	0
hsa-miR-421	1.63622425	0
hsa-miR-1308	2.2919085	0
hsa-miR-183	2.5195705	0
hsa-miR-224	2.590309875	0
hsa-miR-18a	2.830789875	0
hsa-miR-18b	2.862260875	0
M *vs* N DEMs		
hsa-miR-215	-4.0958715	0
hsa-miR-195	-3.200875	0
hsa-miR-497	-3.154721875	0
hsa-miR-139-5p	-2.717419125	0
hsa-miR-10b	-2.615537125	0
hsa-miR-133a	-2.569697875	0
hsa-miR-375	-2.40409025	0
hsa-miR-138	-2.402280625	0
hsa-miR-422a	-2.323093	0
hsa-miR-143	-2.1387105	0
hsa-miR-145	-2.11873625	0
hsa-miR-30a	-2.046916625	0
hsa-miR-130a	-1.8769065	0
hsa-miR-378*	-1.819690375	0
hsa-miR-378	-1.791166375	0

hsa-miR-381	-1.312789	0
hsa-miR-140-3p	-1.261712125	0
hsa-miR-342-5p	-1.180007875	0
hsa-miR-20a	1.004735125	0
hsa-miR-93	1.043093625	0
hsa-miR-210	1.0654855	0
hsa-miR-18a*	1.0906075	0
hsa-miR-106a	1.11549275	0
hsa-miR-27a*	1.147799375	0
hsa-miR-92a-1*	1.198501875	0
hsa-miR-17	1.238445	0
hsa-miR-29b-1*	1.473451375	0
hsa-miR-885-5p	1.487745625	0
hsa-miR-106b*	1.77119125	0
hsa-miR-181d	2.02541625	0
hsa-miR-1246	2.284496125	0
hsa-miR-18b	2.287867	0
hsa-miR-183	2.604235875	0
hsa-miR-18a	2.615767875	0
hsa-miR-552	2.622208375	0
hsa-miR-503	3.0354695	0
hsa-miR-182	3.0650235	0
hsa-miR-122	9.42885325	0
M *vs* T DEMs		
hsa-miR-139-5p	1.125679	0
hsa-miR-210	1.597213875	0
hsa-miR-150	1.67365425	0
hsa-miR-100	2.444512125	0
hsa-miR-122	9.23824025	0

Supplementary Table S3. Survival analysis results obtained considering tumor and liver metastasis samples and corresponding patients.

Tumor samples						
miRNA	Hz	Robust SE	z	P>|z|	[95% Conf. Interval]	
miR-21	0.740697	0.0820238	-2.71	0.007	0.596183	0.9202407
miR-30a	1.523426	0.2439306	-0.57	0.009	1.113082	2.085046
miR-100	1.445654	0.2091773	-0.4	0.011	1.088681	1.919676
miR-1395p	1.302943	0.1430296	2.41	0.016	1.050716	1.615718
miR-125b	1.497941	0.2604764	2.32	0.02	1.065319	2.10625
miR-10b	1.445619	0.2155776	2.29	0.022	1.051186	1.908821
miR-182	0.755852	0.0962479	-2.2	0.028	0.5889083	0.970122
miR-497	1.340884	0.1870204	2.1	0.035	1.020163	1.762434
miR-375	1.249903	0.1368768	2.04	0.042	1.008466	1.549143
miR-143	1.208842	0.1363067	1.68	0.093	0.9691473	1.50782
miR-8855p	1.453348	0.3501852	1.55	0.121	0.9063005	2.330597
miR-18b	1.278434	0.2100506	1.5	0.135	0.9264525	1.764141
miR-422a	1.280228	0.2171003	1.46	0.145	0.9182084	1.784981
miR-195	1.222812	0.1835377	1.34	0.18	0.9111687	1.641046
miR-215	1.14874	0.1233377	1.29	0.197	0.9307437	1.417795
miR-138	1.16255	0.1496256	1.17	0.242	0.9033544	1.496116
miR-145	1.194616	0.2076925	1.02	0.306	0.8496513	1.679638
miR-194	1.175256	0.2058032	0.92	0.356	0.8338262	1.656492
miR-150	1.111189	0.1418009	0.83	0.409	0.8652962	1.426959
miR-378*	1.146621	0.192162	0.82	0.414	0.8255953	1.592475
miR-122	1.04267	0.0591751	0.74	0.462	0.9329066	1.165348
miR-378	1.119906	0.2094414	0.61	0.545	0.776234	1.615738
miR-210	1.027765	0.1769322	0.16	0.874	0.733428	1.440225
miR-1246	1.029083	0.202784	0.15	0.884	0.6993882	1.514196
miR-18a	0.967623	0.2198332	-0.14	0.885	0.6199017	1.510393
miR-183	1.017753	0.1402613	0.13	0.898	0.7768446	1.333369
Metastatic samples						
miRNA	Hz	Robust SE	z	P>|z|	[95% Conf. Interval]	
miR-18b	1.215287	0.0951059	2.49	0.013	1.042475	1.416746
miR-21	0.863168	0.0596274	-2.13	0.033	0.7538671	0.9883171
miR-10b	1.187348	0.0961336	2.12	0.034	1.013119	1.391539
miR-1395p	1.133919	0.0703489	2.03	0.043	1.004091	1.280533
miR-375	1.136724	0.076113	1.91	0.056	0.9969192	1.296134
miR-100	1.118093	0.0745839	1.67	0.094	0.9810646	1.274262
miR-125b	1.124821	0.0966557	1.37	0.171	0.950473	1.33115
miR-194	1.134437	0.1374286	1.04	0.298	0.8946717	1.438458
miR-8855p	1.135833	0.1405195	1.03	0.303	0.8912677	1.447508
miR-183	1.073346	0.0779309	0.97	0.33	0.9309742	1.23749
miR-182	0.942022	0.0874513	-0.64	0.52	0.7853097	1.130007
miR-422a	1.069758	0.1132576	0.64	0.524	0.8692949	1.31645

miR-138	1.052881	0.0961541	0.56	0.573	0.8803255	1.259259
miR-143	1.041168	0.0763013	0.55	0.582	0.9018637	1.201989
miR-18a	1.057718	0.1472312	0.4	0.687	0.8051656	1.389487
miR-497	0.972728	0.0726683	-0.37	0.711	0.8402367	1.12611
miR-215	1.019475	0.0588018	0.33	0.738	0.9105018	1.141492
miR-195	0.983994	0.0615923	-0.26	0.797	0.8703861	1.11243
miR-150	1.01402	0.0625668	0.23	0.821	0.8985161	1.144372
miR-378*	0.981605	0.1016665	-0.18	0.858	0.8012651	1.202533
miR-210	0.982149	0.1214311	-0.15	0.884	0.7707904	1.251464
miR-145	1.010772	0.1123965	0.1	0.923	0.8128315	1.256914
miR-378	1.012747	0.1341352	0.1	0.924	0.7811995	1.312925
miR-30a	1.008267	0.0876904	0.09	0.925	0.8502475	1.195655
miR-1246	0.992936	0.0831854	-0.08	0.933	0.8425773	1.170125
miR-122	0.997429	0.0314328	-0.08	0.935	0.937686	1.060979

Supplementary Table S4. KEGG pathways significantly modulated in T *vs* N and in T *vs* M comparisons. For each comparison, significantly modulated pathways were identified considering expression profiles of all genes being supported targets of DEMs and considering only the subset of genes being supported targets of DEMs and differentially expressed in the same contrast. In bold, miRNAs and genes in common for the two analyses.

KEGG ID	Pathway	T *vs* N DEMs and supported target genes				T *vs* N DEMs and supported target T vs N DEGs			
		miRNA	Sign	Gene	Sign	miRNA	Sign	Gene	Sign
4110	Cell cycle	hsa-miR-182	up	CDKN2B	down	**hsa-miR-195**	down	**CDC25B**	up
		hsa-miR-100	down	ATR	up	**hsa-miR-145**	down	**MYC**	up
		hsa-miR-497	down	CCND1	up			**PRKDC**	up
		**hsa-miR-195**	down	CCNB1	up				
		hsa-miR-30a	down	BUB1	up				
		**hsa-miR-145**	down	CDC25A	up				
		hsa-miR-125b	down	**CDC25B**	up				
		hsa-miR-139-5p	down	CDC25C	up				
		hsa-miR-378	down	E2F3	up				
		hsa-miR-378*	down	MCM2	up				
		hsa-miR-422a	down	**MYC**	up				
				**PRKDC**	up				
				YWHAG	up				
				CDC23	up				
				DBF4	up				
				ORC6L	up				
				ANAPC7	up				
				ANAPC1	up				
230	Purine metabolism	hsa-miR-422a	down	**ENTPD5**	down	**hsa-miR-150**	down	**NME1**	up
		hsa-miR-378	down	PDE1C	down	**hsa-miR-182**	up	**ENTPD5**	down
		**hsa-miR-182**	up	PDE4D	down	**hsa-miR-183**	up	**PAPSS2**	down
		hsa-miR-10b	down	PDE7B	down			**PDE7B**	down
		**hsa-miR-150**	down	**PAPSS2**	down			**NM23A**	up
		**hsa-miR-183**	up	**PDE7B**	up				
		hsa-miR-1246	up	AMPD2	up				
		hsa-miR-145	down	GART	up				
		hsa-miR-138	down	**NME1**	up				
		hsa-miR-125b	down	**NM23A**	up				
		hsa-miR-30a	down	PFAS	up				
		hsa-miR-195	down	PKM2	up				
		hsa-miR-497	down	POLR2D	up				
		hsa-miR-139-5p	down	PPAT	up				
				RRM2	up				
				POLR1A	up				
				POLR1B	up				
				PNPT1	up				
240	Pyrimidine metabolism	hsa-miR-182	up	ENTPD5	down				
		hsa-miR-143*	down	CAD	up				
		hsa-miR-125b	down	CTPS	up				
		hsa-miR-378	down	DTYMK	up				
		hsa-miR-422a	down	NM23A	up				
		hsa-miR-150	down	POLR2D	up				
		hsa-miR-30a	down	RRM2	up				
		hsa-miR-195	down	UCK2	up				
		hsa-miR-497	down	UMPS	up				
		hsa-miR-145	down	POLR1A	up				
		hsa-miR-138	down	POLR1B	up				
		hsa-miR-139-5p	down	PNPT1	up				

61	Fatty acid biosynthesis	hsa-miR-182<br>hsa-miR-378<br>hsa-miR-139-5p<br>hsa-miR-145<br>hsa-miR-497<br>hsa-miR-195	up<br>down<br>down<br>down<br>down<br>down	ACACB<br>ACACA<br>FASN	down<br>up<br>up				
4914	Progesterone-mediated oocyte maturation	hsa-miR-21<br>hsa-miR-182<br>hsa-miR-30a<br>hsa-miR-195<br>hsa-miR-497<br>hsa-miR-100<br>hsa-miR-194*<br>hsa-miR-378*<br>hsa-miR-125b<br>hsa-miR-139-5p	up<br>up<br>down<br>down<br>down<br>down<br>down<br>down<br>down<br>down	PIK3R1<br>PRKACB<br>BUB1<br>CCNB1<br>CDC25A<br>CDC25B<br>CDC25C<br>HSP90A<br>B1<br>CDC23<br>ANAPC7<br>ANAPC1	down<br>down<br>up<br>up<br>up<br>up<br>up<br>up<br>up<br>up<br>up				
4115	p53 signaling pathway	hsa-miR-182<br>hsa-miR-183<br>hsa-miR-30a<br>hsa-miR-195<br>hsa-miR-497<br>hsa-miR-145<br>hsa-miR-194*<br>hsa-miR-378*<br>hsa-miR-125b<br>hsa-miR-139-5p	up<br>up<br>down<br>down<br>down<br>down<br>down<br>down<br>down<br>down	FAS<br>ATR<br>CCND1<br>CCNB1<br>BID<br>SERPINE<br>1<br>RRM2<br>SHISA5<br>GTSE1	down<br>up<br>up<br>up<br>up<br>up<br>up<br>up<br>up				
450	Selenoamino acid metabolism	**hsa-miR-183**<br>**hsa-miR-182**<br>hsa-miR-145<br>hsa-miR-138<br>hsa-miR-30a	up<br>up<br>down<br>down<br>down	**PAPSS2**<br>**AHCYL2**<br>MARS<br>MARS2<br>WBSCR2<br>2	down<br>down<br>up<br>up<br>up	**hsa-miR-183**<br>**hsa-miR-182**	up<br>up	**PAPSS2**<br>**AHCYL2**	down<br>down
970	Aminoacyl-tRNA biosynthesis	hsa-miR-145<br>hsa-miR-150<br>hsa-miR-195<br>hsa-miR-497<br>hsa-miR-138<br>hsa-miR-30a	down<br>down<br>down<br>down<br>down<br>down	AARS<br>IARS<br>KARS<br>MARS<br>TARS2<br>MARS2	up<br>up<br>up<br>up<br>up<br>up				
5222	Small cell lung cancer	hsa-miR-21<br>hsa-miR-182<br>hsa-miR-497<br>hsa-miR-195<br>hsa-miR-150<br>hsa-miR-194*<br>hsa-miR-30a<br>hsa-miR-125b<br>hsa-miR-139-5p<br>hsa-miR-145<br>hsa-miR-378*	up<br>up<br>down<br>down<br>down<br>down<br>down<br>down<br>down<br>down<br>down	PIK3R1<br>CDKN2B<br>CCDN1<br>CKS1B<br>COL4A1<br>E2F3<br>MYC<br>RELA<br>ITGA2	down<br>down<br>up<br>up<br>up<br>up<br>up<br>up<br>up				

5200	Pathways in cancer	**hsa-miR-182**<br>hsa-miR-18a<br>**hsa-miR-1246**<br>hsa-miR-21<br>**hsa-miR-145**<br>**hsa-miR-195**<br>**hsa-miR-497**<br>**hsa-miR-30a**<br>hsa-miR-194*<br>hsa-miR-150<br>hsa-miR-125b<br>**hsa-miR-139-**<br>**5p**<br>hsa-miR-378*<br>**hsa-miR-422a**<br>**hsa-miR-378**<br>hsa-miR-138<br>hsa-miR-183	up<br>up<br>up<br>up<br>down<br>down<br>down<br>down<br>down<br>down<br>down<br>down<br>down<br>down<br>down<br>down<br>up	FAS<br>BMP2<br>CDKN2B<br>GLI3<br>**PDGFRA**<br>PIK3R1<br>PLD1<br>SOS2<br>BIRC5<br>CCDN1<br>BCR<br>BID<br>RUNX1<br>CKS1B<br>COL4A1<br>E2F3<br>HSP90A<br>B1<br>**ITGA2**<br>**MYC**<br>PLCG1<br>RELA<br>**SLC2A1**<br>VEGFA<br>**AXIN2**	down<br>down<br>down<br>down<br>**down**<br>down<br>down<br>down<br>up<br>up<br>up<br>up<br>up<br>up<br>up<br>up<br>up<br>**up**<br>**up**<br>up<br>up<br>**up**<br>up<br>**up**	**hsa-miR-1246**<br>**hsa-miR-182**<br>**hsa-miR-497**<br>**hsa-miR-195**<br>**hsa-miR-139-5p**<br>**hsa-miR-145**<br>**hsa-miR-30a**<br>**hsa-miR-378**<br>**hsa-miR-422a**	up<br>up<br>down<br>down<br>down<br>down<br>down<br>down<br>down	**PDGFRA**<br>**ITGA2**<br>**MYC**<br>**SLC2A1**<br>**AXIN2**	down<br>up<br>up<br>up<br>up
4210	Apoptosis	hsa-miR-182<br>hsa-miR-183<br>hsa-miR-21<br>hsa-miR-30a<br>hsa-miR-195<br>hsa-miR-21<br>hsa-miR-497<br>hsa-miR-378*<br>hsa-miR-1246	up<br>up<br>up<br>down<br>down<br>up<br>down<br>down<br>up	FAS<br>PIK3R1<br>PRKACB<br>TNFRSF1<br>A<br>TNFSF10<br>BID<br>IL1RAP<br>IRAK2<br>RELA	Down<br>down<br>down<br>down<br>down<br>up<br>up<br>up<br>up				
3022	Basal transcription factors	hsa-miR-30a<br>hsa-miR-422a<br>hsa-miR-378<br>hsa-miR-125b<br>hsa-miR-378*	down<br>down<br>down<br>down<br>down	GTF2E1<br>GTF2I<br>TAF4<br>GTF2IRD<br>1<br>TAF5L	up<br>up<br>up<br>up<br>up				
5220	Chronic myeloid leukemia	hsa-miR-21<br>hsa-miR-182<br>hsa-miR-1246<br>hsa-miR-497<br>hsa-miR-195<br>hsa-miR-194*<br>hsa-miR-30a<br>hsa-miR-125b<br>hsa-miR-145<br>hsa-miR-378*	up<br>up<br>up<br>down<br>down<br>down<br>down<br>down<br>down<br>down	PIK3R1<br>SOS2<br>CCND1<br>BCR<br>RUNX1<br>E2F3<br>MYC<br>RELA	down<br>down<br>up<br>up<br>up<br>up<br>up<br>up				
30	Pentose phosphate pathway	hsa-miR-378*<br>hsa-miR-150<br>hsa-miR-422a<br>hsa-miR-139-5p<br>hsa-miR-30a	down<br>down<br>down<br>down<br>down	GPI<br>TALDO1<br>TKT<br>RPIA	up<br>up<br>up<br>up				
630	Glyoxylate and dicarboxylate metabolism	hsa-miR-378<br>hsa-miR-145<br>hsa-miR-30a<br>hsa-miR-138	down<br>down<br>down<br>down	MTHFD1<br>MTHFD1<br>L<br>AFMID	up<br>up<br>up				

4114	Oocyte meiosis	hsa-miR-30a<br>hsa-miR-195<br>hsa-miR-145<br>hsa-miR-497<br>hsa-miR-378*<br>hsa-miR-125b<br>hsa-miR-139-5p<br>hsa-miR-182<br>hsa-miR-18a	down<br>down<br>down<br>down<br>down<br>down<br>down<br>up<br>up	BUB1<br>CCNB1<br>CDC25C<br>PPP2R1A<br>YWHAG<br>CDC23<br>ANAPC7<br>ANAPC1<br>ITPR1<br>PRKACB	up<br>up<br>up<br>up<br>up<br>up<br>up<br>up<br>down<br>down				
100	Steroid biosynthesis	hsa-miR-139-5p<br>hsa-miR-145	down<br>down	DHCR7<br>NSDHL<br>HS17B7	up<br>up<br>up				
670	One carbon pool by folate	hsa-miR-10b<br>hsa-miR-30a<br>hsa-miR-138	down<br>down<br>down	GART<br>MTHFD1<br>MTHFD1<br>L	up<br>up<br>up				
5219	Bladder cancer	hsa-miR-497<br>hsa-miR-30a<br>hsa-miR-125b<br>hsa-miR-195<br>hsa-miR-145<br>hsa-miR-138<br>hsa-miR-182	down<br>down<br>down<br>down<br>down<br>down<br>up	CCND1<br>E2F3<br>MYC<br>VEGFA<br>RPS6KA<br>5	up<br>up<br>up<br>up<br>down				
4310	Wnt signaling pathway					hsa-miR-145<br>hsa-miR-497<br>hsa-miR-195<br>hsa-miR-143*<br>hsa-miR-182	down<br>down<br>down<br>up<br>down	MYC<br>AXIN2<br>RUVBL1<br>PRKACB	up<br>up<br>up<br>down
5210	Colorectal cancer					hsa-miR-1246<br>hsa-miR-182<br>hsa-miR-145<br>hsa-miR-497<br>hsa-miR-195	up<br>up<br>down<br>down<br>down	PDGFRA<br>MYC<br>AXIN2	up<br>down<br>down
750	Vitamin B6 metabolism					hsa-miR-195<br>hsa-miR-497<br>hsa-miR-145<br>hsa-miR-30a	down<br>down<br>down<br>down	PSAT1	up<br>up<br>up<br>down
5213	Endometrial cancer					hsa-miR-145<br>hsa-miR-497<br>hsa-miR-195	down<br>down<br>down	MYC<br>AXIN2	up<br>up

KEGG ID	Pathway	M *vs* T DEMs and supported target genes				M *vs* T DEMs and supported target M *vs* T DEGs			
		miRNA	Sign	Gene	Sign	miRNA	Sign	Gene	Sign
230	Purine metabolism	hsa-miR-10b<br>hsa-miR-122	down<br>up	GART<br>PDE1C	up<br>down				
280	Valine, leucine and isoleucine degradation	hsa-miR-10b	down	MCCC2	up				
670	One carbon pool by folate	hsa-miR-10b	down	GART	up				
